# Comparison of adverse events between video and direct laryngoscopes for tracheal intubations in emergency department and ICU patients–a systematic review and meta-analysis

**DOI:** 10.1186/s13049-020-0702-7

**Published:** 2020-02-07

**Authors:** Jia Jiang, Na Kang, Bo Li, An-Shi Wu, Fu-Shan Xue

**Affiliations:** 1 Department of Anesthesiology, Beijing Chaoyang Hospital, Capital Medical University, Beijing, 100020 China; 2 Beijing Hospital of Traditional Chinese Medicine, Capital Medical University, Beijing Institute of Traditional Chinese Medicine, Beijing, 100010 China; 3 Department of Anesthesiology, Beijing Friendship Hospital, Capital Medical University, Beijing, 100050 China

**Keywords:** Airway management, Laryngoscope, Tracheal intubation, Randomized controlled trial, Observational study

## Abstract

**Objective:**

This systematic review and meta-analysis was designed to determine whether video laryngoscope (VL) compared with direct laryngoscope (DL) could reduce the occurrence of adverse events associated with tracheal intubation in the emergency and ICU patients.

**Methods:**

The current issue of Cochrane Central Register of Controlled Trials, PubMed, EMBASE, and Web of Science (from database inception to October 30, 2018) were searched. The RCTs, quasi-RCTs, observational studies comparing VL and DL for tracheal intubation in emergency or ICU patients and reporting the rates of adverse events were included. The primary outcome was the rate of esophageal intubation (EI). Review Manager 5.3 software was used to perform the pooled analysis and assess the risk of bias for each eligible RCT. The ACROBAT-NRSi Cochrane Risk of Bias Tool was applied to assess the risk of bias for each eligible observational study.

**Results:**

Twenty-three studies (13,117 patients) were included in the review for data extraction. Pooled analysis showed a lower rate of EI by using VL (relative risk [RR], 0.24; *P* < 0.01; high-quality evidence for RCTs and very low-quality evidence for observational studies). Subgroup analyses based on the type of studies, whether a cardiopulmonary resuscitation study, or operators’ expertise showed a similar lower rate of EI by using VL compared with DL in all subgroups (*P* < 0.01) except for experienced operators (RR, 0.44; *P* = 0.09). There were no significant differences between devices for other adverse events (*P* > 0.05), except for a lower incidence of hypoxemia when intubation was performed with VL by inexperienced operators (*P* = 0.03).

**Conclusions:**

Based on the results of this analysis, we conclude that compared with DL, VL can reduce the risk of EI during tracheal intubation in the emergency and ICU patients, but does not provide significant benefits on other adverse events associated with tracheal intubation.

## Introduction

Tracheal intubation is a primary lifesaving procedure for emergency department (ED) or intensive care unit (ICU) patients associated with respiratory dysfunction or decreased airway protection. Under the urgent situation, however, airway management can be challenging due to decompensated cardiopulmonary physiology, inadequate provision of tools and skilled staffs, unfasted state, simultaneous performance of cardiopulmonary resuscitation (CPR) or other medical procedures, difficult access to the patient head, and various anatomic features of difficult airways [1]. It has been reported that failure rate of urgent tracheal intubations in the ED and ICU is significantly higher compared to tracheal intubations performed in the operating room [2]. Furthermore, the results of the Fourth National Audit Project of the Royal College of Anaesthetists and the Difficult Airway Society shows that at least one in four major adverse airway events in a hospital are likely to occur in the ED or ICU and adverse airway events leading to death or brain damage are approximately 30-fold and 60-fold more frequent in the ICU and ED than in the operating room [1]. Thus, the strategies or alternative methods to reduce the occurrence of adverse events associated with urgent tracheal intubations in the ED and ICU patients are needed.

Traditionally, tracheal intubation is performed using direct laryngoscope (DL), which requires the alignment of the oral, pharyngeal and laryngeal axes to allow direct visualization of the glottis. Thus, tracheal intubation with DL is generally regarded as a difficult skill to acquire and maintain. Video laryngoscope (VL) is a new device that contains a miniaturized camera at the blade tip to indirectly visualize the glottis. Because of proven advantages of a fast learning curve, an improved laryngeal visualization and an increased success rate, VL has been widely used for tracheal intubation in emergency and critical situations [3–22]. There have been several meta-analyses comparing VL with DL for tracheal intubation in the ED and ICU patients, but they have mainly focused on the success rate of tracheal intubation and provided different outcomes [23–29]. Most important, it is unclear whether the use of VL can reduce the adverse events associated with tracheal intubations in the ED and ICU patients. Thus, this systematic review and meta-analysis was carried out to determine whether VL compared with DL could reduce the adverse events associated tracheal intubation in the ED and ICU patients.

## Materials and methods

This systematic review and meta-analysis was conducted following the recommendations of The Cochrane Handbook for Systematic Reviews of Interventions and reported according to the PRISMA statement [30] (www.prisma-statement.org). The protocol was registered on the PROSPERO (http://www.crd. york.ac.uk/PROSPERO, ID: CRD42018100562).

### Data sources and search strategy

The current issue of the Cochrane Central Register of Controlled Trials (CENTRAL; 2017, Issue 9), PubMed (1946 to October 30th, 2018), EMBASE (1974 to October 30th, 2018), and Web of Science (1900 to October 30th, 2018) were searched. Study authors were mailed for any useful information. The reference lists of all eligible trials and reviews were screened for additional citations. No language restriction was imposed. The search strategies of the four electronic databases were provided in Table 1.

**Table 1:** Search strategy for four databases

Database	Search Strategy
PubMed	(((((((ED[Title/Abstract]) OR critical*[Title/Abstract]) OR urgent[Title/Abstract]) OR ICU[Title/Abstract]) OR emergen*[Title/Abstract])) AND (((((((((((((“King Vision”[Title/Abstract]) OR McGrath[Title/Abstract]) OR Glidescope[Title/Abstract]) OR C-MAC[Title/Abstract]) OR Airtraq[Title/Abstract]) OR “Airway Scope”[Title/Abstract]) OR “Pentax AWS”[Title/Abstract]) OR “TruView PCD”[Title/Abstract]) OR “Storz DCI”[Title/Abstract]) OR BERCI[Title/Abstract]) OR “video laryngoscopy”) OR “video laryngoscope”[Title/Abstract]) OR videolaryngoscop*[Title/Abstract])
Embase	(‘videolaryngoscopy’:ab,ti OR ‘videolaryngoscope’:ab,ti OR ‘video laryngoscopy’:ab,ti OR ‘video laryngoscope’:ab,ti OR ‘king vision’:ab,ti OR glidescope:ab,ti OR mcgrath:ab,ti OR ‘c mac’:ab,ti OR airtraq:ab,ti OR ‘airway scope’:ab,ti OR ‘pentax aws’:ab,ti OR truview:ab,ti OR ‘storz dci’:ab,ti OR berci:ab,ti) AND (emergen*:ab,ti,kw OR critical*:ab,kw,ti OR icu:ab,kw,ti OR ed.:ab,ti,kw) AND (‘case report’/de OR ‘clinical article’/de OR ‘clinical trial’/de OR ‘comparative study’/de OR ‘controlled clinical trial’/de OR ‘controlled study’/de OR ‘crossover procedure’/de OR ‘human’/de OR ‘human experiment’/de OR ‘intermethod comparison’/de OR ‘major clinical study’/de OR ‘meta analysis’/de OR ‘multicenter study’/de OR ‘observational study’/de OR ‘prospective study’/de OR ‘randomized controlled trial’/de OR ‘randomized controlled trial (topic)’/de OR ‘retrospective study’/de OR ‘total quality management’/de)
Cochrane Central Register of Controlled Trials	‘videolaryngoscopy or videolaryngoscope or videolaryngoscopic or videolaryngoscopes or “video laryngoscopy” or “video laryngoscope” or “video laryngoscopic” or “video laryngoscopes” or “King Vision” or McGrath or Glidescope or C-MAC OR Airtraq or “Airway Scope” or “Pentax AWS” or “TruView PCD” or “Storz DCI” or BERCI in Title, Abstract, Keywords and emergent or critical or ICU or urgent in Title, Abstract, Keywords’
Web of Science	TI = (videolaryngoscop* OR “video laryngoscop*” OR “King Vision” OR Glidescope OR McGrath OR C-MAC OR Airtraq OR “Airway Scope” OR “Pentax AWS” OR “TruView PCD” OR “Storz DCI” OR “BERCI” OR “AP Advance”) AND TS = (urgent or ICU or ED or critical* or emergen*) Index = SCI-EXPANDED, SSCI, A&HCL, ESCI Timespan = All years

### Eligibility criteria

RCTs, quasi-RCTs, and observational (prospective or retrospective) studies comparing VL and DL and reporting the adverse events of tracheal intubations in the ED and ICU patients were included. Conference abstracts with available data were also included. Studies in which VL or DL was used as a rescue device were excluded. Pre-hospital study, manikin study, cadaver study, simulated study, or case reports were also excluded. Participants were in-hospital non-surgical patients needing urgent tracheal intubations in the ED and ICU. Patients with a suspected laryngeal trauma or an extensive maxillofacial injury requiring an immediate surgical airway, supraglottic airway, or awake fiberoptic intubation were excluded. Patients in the intervention group used a VL. When the C-MAC or McGrath MAC laryngoscope was used for DL by inexperienced operator, the attempt was considered a VL, regardless of whether the operator looked at the monitor. In this case, the supervising attending physician was able to view the video monitor and assist with the tracheal intubation [5]. For patients in the control group, a DL was used. Optimizing maneuvers such as the external laryngeal pressure, the use of intubation stylet or introducer, could be initiated at the discretion of the operator.

### Study selection and data extraction

The titles and abstracts were independently screened by two study authors (J.J.; N.K.). After retrieving the full-texts or conference abstract of any potentially relevant studies, their eligibilities were determined. Any disagreements between the two review authors were resolved by discussion with other authors until a consensus was obtained. A PRISMA flow diagram was completed to record the selection process in detail [31].

Data was independently extracted by two review authors (J.J.; N.K.). All the outcomes were dichotomous variables, the number of events occurred and the sample sizes were extracted. Any disagreement on data extraction was resolved by discussion with a third author (F.S.X.) until a consensus was reached.

### Primary and secondary outcomes

The esophageal intubation (EI) is rare, but is one of mostly severe adverse events associated with tracheal intubation in the ICU and ED patients [3]. Thus, the rate of EI was used as primary outcome. If the rates of EI at the first-attempt and at any attempt were given, the rate of EI at the first-attempt was only used. The secondary outcomes were incidences of hypoxemia (SpO_2_ < 90%) and severe hypoxemia (SpO_2_ < 80%) during the intubation procedure, incidence of aspiration (any witnessed aspiration of gastric contents during the intubation attempt or defined by the original author), incidence of new-onset cardiac arrest (during or immediately after intubation), short-term (within 24 h) and long-term (28 d or in-hospital) all-causes mortality.

### Risk of bias assessment

The risk of bias for each eligible study was independently assessed by two review authors (J.J. and N.K.). For RCTs (including quasi-RCT), the Cochrane Collaboration’s tool was used to assess the risk of bias [32]. Each of the seven domains, such as random sequence generation, allocation concealment, blinding of participants and personnels, blinding of outcome assessment, incomplete outcome data, selective reporting, and other biases, was judged as either low, high, or unclear. If all domains were assigned to the “low risk” of bias category, the study was classified as “low risk”; if one or more domains were assigned to the “unclear risk” of bias category, the study was classified as “unclear risk”; if one or more domains were assigned to the high risk of bias category, the study was classified as “high risk” [32]. For observational studies, the ACROBAT-NRSi Cochrane Risk of Bias Tool was used [33]. Each of the seven domains, such as confounding, selection of participants, classification of interventions, deviations from intended interventions, missing data, measurement outcomes, and selection of the reported outcomes, was judged as either low, moderate, severe, or unclear. The possible confounding domains are “experience of operators, difficult airways, number of patients with CPR, and the use of neuromuscular blockades”. No co-interventions were considered. An overall judgement of the risk of bias for each study was reached as low, moderate, serious, critical or no information on the risk of bias. Reporting bias was also assessed by using funnel plot if the result of primary outcome was from at least 10 trials [34].

The criteria of the Grading of Recommendations Assessment, Development and Evaluation (GRADE) system (study limitations, consistency of effect, imprecision, indirectness, and publication bias) were applied to assess the quality of evidence associated with all outcomes [35, 36]. Then a “Grade evidence profile” table was developed by using the GRADE software (www.guidelinedevelopment.org) to rate these outcomes as high, moderate, low, or very low quality. Since different types of studies were involved in this review, the quality of the evidence was assessed for the RCTs (including quasi-RCT) and non-RCTs, respectively. The quality of evidence was downgraded by one or two levels when serious or very serious deficiencies were considered in these criteria.

Trial sequential analysis (TSA) was performed for all adverse events from RCTs [37, 38]. The information size required was calculated to provide an estimate of how many more patients would be required to make a reliable conclusion. A conventional calculation for sample size estimation, with conventional values for α and β error (0.05 and 0.20), low bias-based relative risk reduction, the incidence in control arm, and a model variance-based heterogeneity correction was used.

### Statistical analysis

The study authors of the original report were contacted for important missing statistics. For the participants missing due to dropout, if “missing at random”, analysis was performed based on the available data; if not, an available case analysis was performed and the potential bias was explained in discussion section. If a study did not mention withdrawals, no drop-out was assumed.

Both relative risk (RR) and 95% confidence interval (CI) were used for dichotomous data. *P* < 0.05 was considered statistically significant. Review Manager was applied to perform the pooled analyses for the outcomes from more than one study. A Chi-squared test with the *I*^2^ statistic (with statistical significance set at the level of two-tailed 0.10) was used to describe the percentage of the total variance across studies from heterogeneity rather than from chance. If *I*^2^ is < 40%, namely there was no statistical heterogeneity among studies, and a fixed-effect model was used; otherwise, a random-effect model was used. For the results that could not be analyzed via meta-analysis, only a qualitative systematic review was planned instead of excluding that study.

Clinical and methodological heterogeneities were considered before performing pooled analysis. In the presence of a statistical heterogeneity or an indication of clinical heterogeneity, subgroup analysis was carried out for all outcomes according to the following possible heterogeneous factors: the type of studies, RCTs or non-RCTs; whether a specialized CPR study; and operators’ expertise: experienced (certificated anesthesiologist, physicians of emergency medical services with no less than three years of clinical experience, physicians performed 50 successful tracheal intubations, or according to the judgment of authors) or inexperienced. If both experienced and inexperienced operators were involved in one study, the sub-grouping of the expertise was determined by the majority part.

## Results

### Characteristic of included studies

**Fig. 1:**
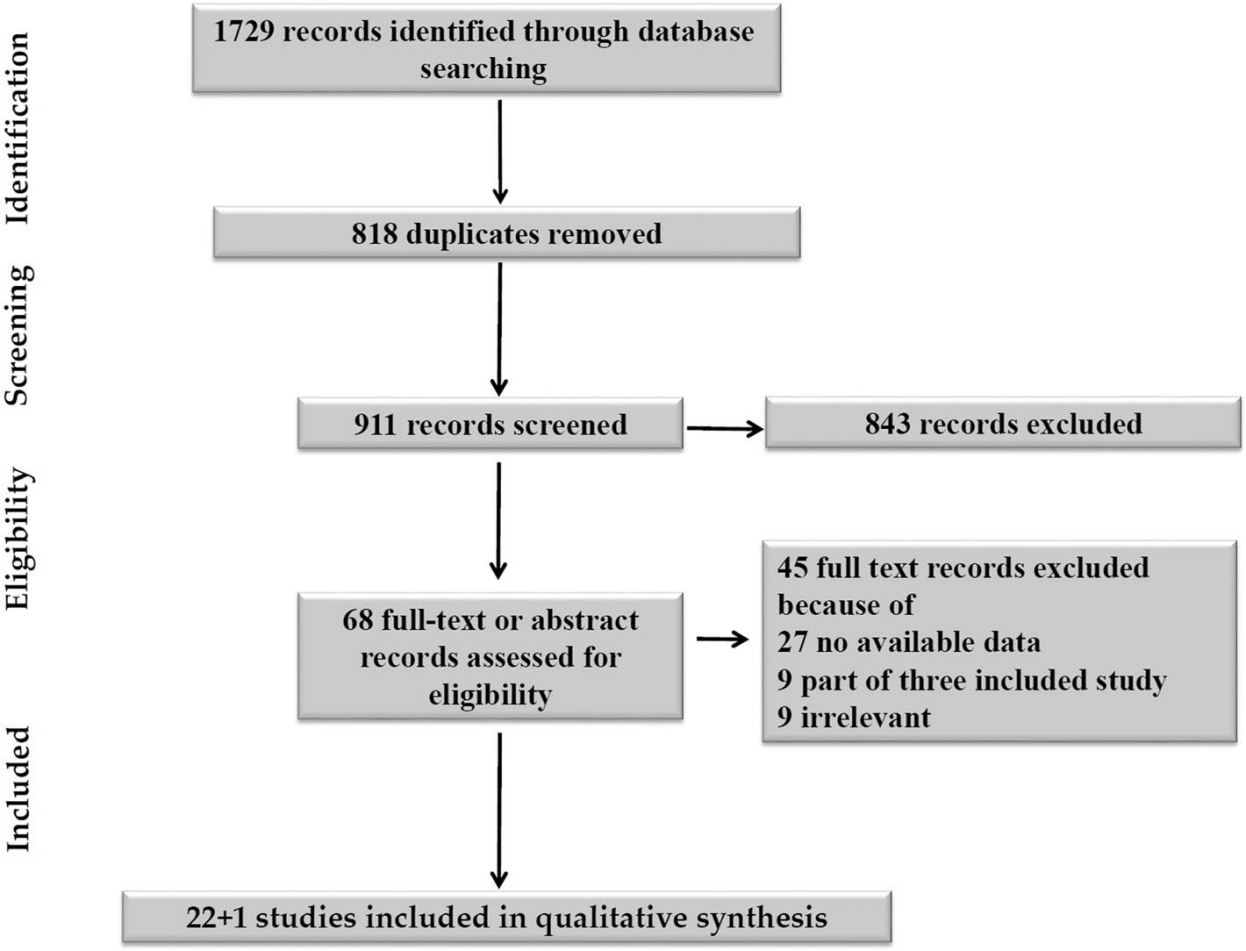
Flow chart of included and excluded studies

Using search strategy, a total of 1729 papers were identified. Of them, 1661 were excluded during title and abstract screening due to duplicates and being irrelevant to our research question. Sixty-eight studies were selected for full-text assessment using inclusion and exclusion criteria. Forty-five studies were further removed because of no available data (*n* = 27), overlapping data sets (*n* = 9), or irrelevant (n = 9). Finally, 23 studies (*n* = 13,117) including one conference abstract were eventually included in the review for data extraction. Authors from three studies [3, 6, 14] were contacted for detailed information on important data, only one of them replied [3]. The flow chart of included and excluded studies is shown in Fig. 1.

The characteristics of included studies are listed in Table 2. Of the 23 included studies, 9 were carried out in the ICU [4, 5, 7, 9, 14, 16, 17, 19, 20], 11 in the ED, one in both the ICU and wards [6], one in general wards [8], and the other in out-of-operating room location [39]; 4 studies enrolled only patients with cardiac arrest [8, 10, 11, 18] and 4 excluded patients with cardiac arrest [16, 19, 21, 22]; 6 studies used a VL with a standard blade (C-MAC) [9, 12, 13, 15, 21, 40], 12 used a VL with an angled blade (GlideScope, McGrath, UEscope) [4, 6, 7, 11, 14, 16, 18–20, 22, 39, 41], and 5 combined several types of VLs (Angled: GlideScope and McGrath; Standard: C-MAC; Channeled: AirwayScope, or KingVision) [3, 5, 8, 10, 17]; tracheal intubation was performed mostly by experienced operators in 7 studies [9, 13, 14, 18, 21, 22, 39], the meeting abstract did not mention the expertise of the operators; Rapid sequence induction (RSI) with sedatives and neuromuscular blockades was chosen as needed in most included studies except for the CPR patients or the studies performed in the CPR patients, 2 studies did not mention the anesthesia induction method [39, 41].

**Table 2:** Characteristics of 23 included studies

Studies	Type of studies	Settings	Number (VL/DL)	Participants	Type of VLs	Experience of operators	Difficult Airway Excluded	Anesthesia
Campagne et al., 2008	Prospective OS	ED	280 (63/210)	Adults	GVL	Unclear	Unknown	Unclear
De Jong et al., 2013	Prospective before–after OS	ICU	210 (140/70)	Adults	McGrath MAC	Most are inexperienced	No	Most RSI
Driver et al., 2016	RCT	ED	198 (103/95)	Adults	C-MAC	Most experienced	No	Most RSI
Driver et al., 2018	OS	ED	823 (428/395)	Adults	C-MAC	Inexperienced	Unclear	Most RSI
Gao et al., 2018	RCT	ICU	163 (81/82)	Adults	UEscope	Experienced	No	Sedatives without any NMBA
Goksu et al., 2016	RCT	ED	150 (75/75)	Age ≥ 16 years	C-MAC	Most inexperienced (ED residents and attending physicians)	No	All used RSI except patients with cardiac arrest.
Griesdale et al., 2012	RCT	ICU	40 (20/20)	Age ≥ 16 years without cardiac arrest. Patients with cervical spine precautions, anticipated difficult intubation, oxygen saturation < 90%, or systolic blood pressure < 80 mmHg despite resuscitation were excluded.	GVL	Inexperienced	Yes	All RSI
Hypes et al., 2016	OS	ICU	809 (673/136)	Nasotracheal intubations, flexible fiberoptic bronchoscopy or supraglottic devices, and intubations performed by medical students or attending physicians were excluded.	GVL, C-MAC, KingVision, and McGrath MAC	Inexperienced	No	Most RSI
Janz et al., 2016	RCT	ICU	150 (74/76)	Adult	McGrath MAC and GVL	Inexperienced	No	All used sedatives and most used NMBAs
Khandelwal et al., 2014	Retrospective	In-hospital, out-of-OR	420 (49/371)	Adult	GVL	Most experienced	No	Unclear
Kim et al., 2016	RCT	ED	140 (71/69)	Adult patients with CPR	GVL	Experienced	No	None
Kory et al., 2013	Retrospective	ICU and medical or surgical wards	138 (78/50)	Not mentioned	GVL	Inexperienced operators	No	NMBAs were not used routinely.
Lakticova et al., 2015	OS	ICU	392 (252/140)	Not mentioned	GVL	Relatively inexperienced	No	Graded intravenous sedation without NMBAs
Lascarrou et al., 2017	RCT	ICU	371 (186/185)	Adult patients without CPR	MaGrath Mac	Most inexperienced	No	All RSI
Lee et al., 2014	Retrospective	General wards	229 (121/108)	Patients in general wards with acute deterioration in clinical status or requiring CPR	GVL or AirwayScope	Inexperienced (50.2%) and experienced (49.8%)	Unclear	Probably none
Noppens et al., 2012	Prospective, before-after study	Anesthetist-lead surgical ICU	230 (117/113)	Adult	C-MAC	Most experienced	No	All used RSI except patients with cardiac arrest.
Okamoto et al., 2018	Multicentre, prospective, OS	ED	3360 (613/2747)	Adult patients with cardiac arrest	C-MAC,McGrath, AirwayScope, and GVL	Most inexperienced	No	Probably none
Park et al., 2015	Historically controlled clinical design	ED	83 (49/34)	All adults out-of-hospital cardiac arrest patients requiring emergency tracheal intubation during CPR in the ED	GVL	Inexperienced	Unclear	Probably none
Sakles et al., 2015	Retrospective	ED	3425 (1895/1530)	All patients in whom intubation attempts were made in the ED by EM residents with a DL or VL.	GVL or C-MAC	Inexperienced	No	Most RSI
Silverberg et al., 2015	quasi-RCT	ICU	117 (57/60)	Patients with a known history of difficult intubation, presence of limited mouth opening, oropharyngeal masses, or swollen tongue, or oxygen saturation less than 92% after bag valve mask ventilation were excluded.	GVL	Inexperienced	Yes	Sedatives were chosen as needed, but NMBA was not used.
Sulser et al., 2016	RCT	ED	147 (74/73)	Adult patients without CPR	C-MAC	Experienced	No	As needed
Vassiliadis et al., 2015	Retrospective	ED	619 (353/266)	ED patients	C-MAC	Most inexperienced	No	Most RSI
Yeatts et al., 2013	RCT	ED	623 (303/320)	CPR patients were excluded.	GVL	Experienced	No	All RSI

VL, videolaryngoscopy; DL, direct laryngoscope; RCT, randomized controlled trial; OS, Observational study; ED, emergency department; EM, emergency medicine; ICU, intensive care unit; OR, operating room; GVL, GlideScope videolarygoscope; CPR, cardiopulmonary resuscitation; RSI, rapid sequence induction; NMBA, neuromuscular blocking agent; LOS, length of stay

### Risk of bias assessment for included studies

Detailed description regarding the risk of bias of the included studies is shown in Table 3. Of the 23 included studies, 9 were RCTs [13–19, 21, 22], one was quasi-RCT [20], and the others were observational studies. The overall risk of bias for most of the included RCTs was rated as low, but the overall risk of bias for the observational studies was rated as moderate or serious mainly due to confounding domains. The funnel plot obtained from primary outcome with its visually symmetrical distribution qualitatively indicated a low risk of publication bias **(**Additional file 1: Fig. S1). The GRADE system showed that the quality of evidence from most RCTs was high or moderate, whereas the quality of evidence from most non-RCTs was very low. The quality of evidence was downgraded manly due to inconsistency from moderate or high level of heterogeneity and imprecision from few participants and few events. The results regarding the quality of evidence for different adverse events were listed in Additional file 2: Table S1.

**Table 3:** Risk of bias assessment of 23 included RCTs and non-RCTs

RCTs	Study Authors	Random sequence generation	Allocation concealment	Blinding of participants and personnel^a^	Blinding of outcome assessment^a^	Incomplete outcome data	Selective reporting	Other bias	Overall
	Driver et al., 2016	Low	Low	Low	Low	Low	Low	Low	Low
	Gao et al., 2018	Unclear	Unclear	Low	Low	Low	Low	Low	Unclear
	Goksu et al., 2016	Low	Low	Low	Low	Low	Low	Low	Low
	Griesdale et al., 2012	Low	Low	Low	Low	Low	Low	Low	Low
	Janz et al., 2016	Low	Low	Low	Low	Low	Low	Low	Low
	Kim et al., 2016	Low	Low^b^	low	low	Low	low	low	Low
	Lascarrou et al., 2017	Low	Low	Low	Low	Low	Low	Low	Low
	Silverberg et al., 2015	High^c^	Unclear	Low	Low	Low	Low	Low	High
	Susler et al., 2016	Low	Low	Low	Low	Low	Low	Low	Low
	Yeatts et al., 2013	Low	Low	Low	Low	High^d^	Low	Low	High
non-RCTs	Study Authors	Confounding	Selection of participants into study	Classification of interventions	Deviations from intended interventions	Missing data	Measurement of outcomes	Selection of reported results	Overall
	Campagne et al., 2008	Unclear	Unclear	Unclear	Unclear	Unclear	Unclear	Unclear	Unclear
	De Jong et al., 2013	Serious^e^	Moderate	Moderate	Moderate	Moderate	Serious^f^	Low	Serious
	Driver et al., 2018	Moderate	Moderate	Moderate	Moderate	Moderate	Low	Low	Moderate
	Hypes et al., 2016	Moderate	Moderate	Moderate	Moderate	Moderate	Moderate	Low	Moderate
	Khandelwal et al., 2014	Moderate	Moderate	Moderate	Moderate	Moderate	Moderate	Low	Moderate
	Kory et al., 2013	Moderate	Moderate	Moderate	Moderate	Moderate	Serious^g^	Low	Serious
	Lakticova et al., 2015	Moderate	Moderate	Moderate	Moderate	Moderate	Moderate	Low	Moderate
	Lee et al., 2014	Serious^e^	Moderate	Moderate	Moderate	Moderate	Moderate	Low	Serious
	Noppens et al., 2012	Serious^e^	Moderate	Moderate	Moderate	Moderate	Moderate	Low	Serious
	Okamoto et al., 2018	Serious ^eh^	Moderate	Moderate	moderate	Moderate	Moderate	Low	Serious
	Park et al., 2015	Moderate	Moderate	Moderate	Moderate	Moderate	Moderate	Low	Moderate
	Sakles et al., 2015	Moderate	Moderate	Moderate	Moderate	Moderate	Serious	Low	Serious
	Vassiliadis et al., 2015	Moderate	Moderate	Moderate	Moderate	Moderate	Serious^f^	Low	Serious

^a^Although all studies did not use blinded method, authors judged that the outcome would not be likely to be influenced as patients were unaware of their grouping and it was impossible for operators to be unaware of the patients’ grouping during intubation process. Moreover, although subjective judgments may bias the results in the absence of blinding, most of our important endpoints are robust; ^**b**^Intubation was required so emergently that a randomization envelope could not be obtained; ^c^An even/odd numbered randomization strategy was used; ^d^There was no reason for missing data provided in this study.

^e^The skill of operators was significantly different between groups; ^f^The analysis was based on the number of intubations rather than the number of patients; ^g^The methods of data collection were different; ^h^Indications of intubation were different between groups

### Rate of EI

Eighteen studies reported the rate of EI [3–7, 9–12, 14, 15, 17–21, 39, 41]; among them, 2 studies reported the rate of EI based on the number of intubations rather than the number of patients [4, 12], one study reported based on the attempts instead of the number of patients [3]. We emailed the original authors for the data based on patients and the initial intubation devices, but the data were not available. After discussion with other authors in our study, we decided to use the current data and do a sensitive analysis by excluding the data from these 3 studies [3, 4, 12].

**Fig. 2:**
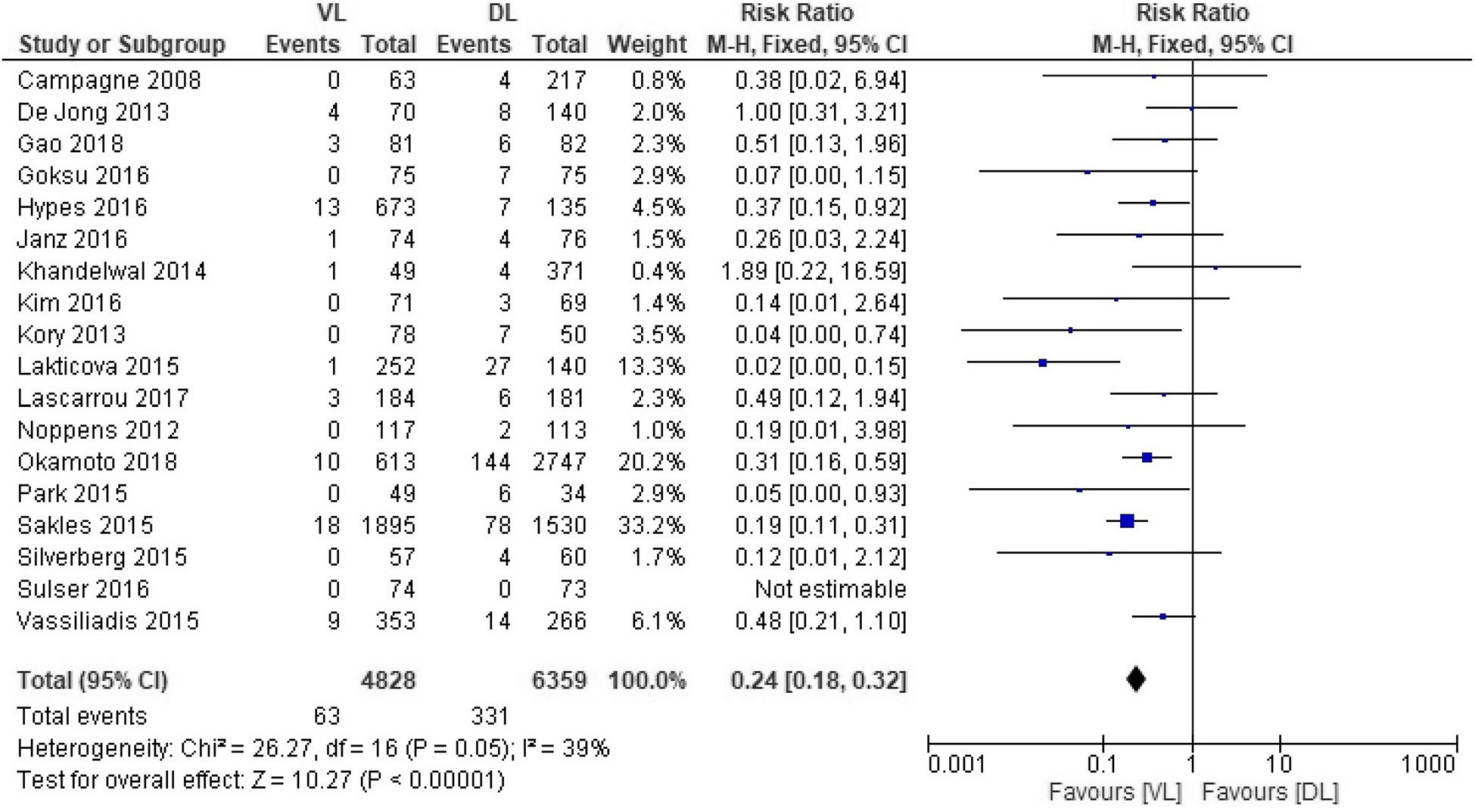
Forest plot for comparison of rate of esophageal intubation based on the type of studies between video laryngoscope (VL) and direct laryngoscope (DL). M-H, Mantel-Haenszel

Pooled analysis showed a significant difference in the rate of EI between VL and DL (RR, 0.24; 95% CI, 0.18–0.32; *n* = 11,187; *P* < 0.01; high-quality evidence for RCTs and very low-quality evidence for observational studies). There was no significant heterogeneity among studies (*P* = 0.05; *I*^2^ = 39%) **(**Additional file 1: Fig. S2). Sensitive analysis by excluding the data from 3 studies mentioned above did not change the pooled result. Subgroup analyses based on the type of studies, whether a CPR study, or operators’ expertise showed a lower rate of EI by using VL compared with DL in all subgroups (*P* < 0.01) except for experienced operators (5 studies; RR, 0.44; 95% CI, 0.17–1.15; *n* = 1100; *P* = 0.09; Fig. 2, Additional file 1: Fig. S3-S4).

### Other adverse events

Results of adverse events including hypoxemia, severe hypoxemia, aspiration, new-onset cardiac arrest, 24 h-mortality, and 28 d-mortality are summarized in Table 4 and Additional file 1: Fig. S5-S19.

**Table 4:** Results of meta-analysis for all adverse events between direct laryngoscope and video laryngoscope

Outcomes	Studies, N	Participants, N	Heterogeneity	Heterogeneity Statistical method	Effect Estimate	*P* values
Rate of EI	18	11,187	*P* = 0.05; *I*^2^ = 39%	RR (M-H, Fixed, 95% CI)	0.24 [0.18, 0.32]	*P* < 0.01
Rate of EI (RCT)	7	1232	*P* = 0.70; *I*^2^ = 0%	RR (M-H, Fixed, 95% CI)	0.27 [0.13, 0.57]	*P* < 0.01
Rate of EI (non-RCT)	11	9955	*P* = 0.01; *I*^2^ = 57%	RR (M-H, Fixed, 95% CI)	0.24 [0.18, 0.32]	*P* < 0.01
Rate of EI (CPR)	4	3723	*P* = 0.44; *I*^2^ = 0%	RR (M-H, Fixed, 95% CI)	0.27 [0.15, 0.49]	*P* < 0.01
Rate of EI (non-CPR)	15	7464	*P* = 0.03; *I*^2^ = 46%	RR (M-H, Fixed, 95% CI)	0.23 [0.17, 0.31]	*P* < 0.01
Rate of EI (experienced)	5	1100	*P* = 0.45; *I*^2^ = 0%	RR (M-H, Fixed, 95% CI)	0.44 [0.17, 1.15]	*P* = 0.09
Rate of EI (inexperienced)	12	9807	*P* = 0.02; *I*^2^ = 50%	RR (M-H, Fixed, 95% CI)	0.23 [0.17, 0.31]	*P* < 0.01
Rate of EI (unknown expertise)	1	280	Not applicable	RR (M-H, Fixed, 95% CI)	0.38 [0.02, 6.94]	*P* = 0.51
Incidence of hypoxemia	4	1548	*P* = 0.35; *I*^2^ = 8%	RR (M-H, Fixed, 95% CI)	0.85 [0.69, 1.05]	*P* = 0.14
Incidence of hypoxemia (RCT)	2	510	*P* = 0.34; *I*^2^ = 0%	RR (M-H, Fixed, 95% CI)	0.91 [0.56, 1.47]	*P* = 0.70
Incidence of hypoxemia (non-RCT)	2	1038	*P* = 0.13; *I*^2^ = 56%	RR (M-H, Fixed, 95% CI)	0.83 [0.66, 1.05]	*P* = 0.13
Incidence of hypoxemia (experienced)	2	393	*P* = 0.70; *I*^2^ = 0%	RR (M-H, Fixed, 95% CI)	1.05 [0.77, 1.43]	*P* = 0.76
Incidence of hypoxemia (inexperienced)	2	1155	*P* = 0.89; *I*^2^ = 0%	RR (M-H, Fixed, 95% CI)	0.71 [0.53, 0.96]	*P* = 0.03
Incidence of severe hypoxemia	8	1739	*P* = 0.26; *I*^2^ = 22%	RR (M-H, Fixed, 95% CI)	1.13 [0.83, 1.52]	*P* = 0.44
Incidence of severe hypoxemia (RCT)	4	787	*P* = 0.18; *I*^2^ = 39%	RR (M-H, Fixed, 95% CI)	1.11 [0.66, 1.87]	*P* = 0.39
Incidence of severe hypoxemia (non-RCT)	4	952	*P* = 0.26; *I*^2^ = 26%	RR (M-H, Fixed, 95% CI)	1.13 [0.78, 1.64]	*P* = 0.52
Incidence of severe hypoxemia (experienced)	2	393	*P* = 0.46; *I*^2^ = 0%	RR (M-H, Fixed, 95% CI)	1.16 [0.62, 2.16]	*P* = 0.65
Incidence of severe hypoxemia (inexperienced)	6	1346	*P* = 0.14; *I*^2^ = 40%	RR (M-H, Fixed, 95% CI)	1.12 [0.79, 1.58]	*P* = 0.53
Incidence of aspiration	13	4634	*P* = 0.98; *I*^2^ = 0%	RR (M-H, Fixed, 95% CI)	0.83 [0.60, 1.16]	*P* = 0.28
Incidence of aspiration (RCT)	7	1751	*P* = 0.79; *I*^2^ = 0%	RR (M-H, Fixed, 95% CI)	0.90 [0.52, 1.58]	*P* = 0.72
Incidence of aspiration (non-RCT)	6	2883	*P* = 0.92; *I*^2^ = 0%	RR (M-H, Fixed, 95% CI)	0.80 [0.53, 1.21]	*P* = 0.28
Incidence of aspiration (CPR)	1	140	Not applicable	RR (M-H, Fixed, 95% CI)	5.58 [0.25, 126.46]	*P* = 0.28
Incidence of aspiration (non-CPR)	13	4494	*P* = 0.97; *I*^2^ = 0%	RR (M-H, Fixed, 95% CI)	0.83 [0.59, 1.16]	*P* = 0.27
Incidence of aspiration (experienced)	6	1769	*P* = 0.88; *I*^2^ = 0%	RR (M-H, Fixed, 95% CI)	0.65 [0.32, 1.32]	*P* = 0.24
Incidence of aspiration (inexperienced)	7	2865	*P* = 0.92; *I*^2^ = 0%	RR (M-H, Fixed, 95% CI)	0.90 [0.61, 1.31]	*P* = 0.57
Incidence of new-onset CA	7	2433	*P* = 0.20; *I*^2^ = 31%	RR (M-H, Fixed, 95% CI)	1.52 [0.63, 3.66]	*P* = 0.35
Incidence of new-onset CA (RCT)	4	795	*P* = 0.56; *I*^2^ = 0%	RR (M-H, Fixed, 95% CI)	3.51 [0.73, 16.92]	*P* = 0.12
Incidence of new-onset CA (non-RCT)	3	1638	*P* = 0.09; *I*^2^ = 58%	RR (M-H, Fixed, 95% CI)	0.86 [0.27, 2.70]	*P* = 0.79
Incidence of new-onset CA (experienced)	1	163	Not applicable	RR (M-H, Fixed, 95% CI)	3.04 [0.13, 73.46]	*P* = 0.49
Incidence of new-onset CA (inexperienced)	6	2270	*P* = 0.14; *I*^2^ = 43%	RR (M-H, Fixed, 95% CI)	1.42 [0.57, 3.56]	*P* = 0.45
24 h-mortality	6	1477	*P* = 0.82; *I*^2^ = 0%	RR (M-H, Fixed, 95% CI)	1.29 [0.98, 1.69]	*P* = 0.07
24 h-mortality (RCT)	3	646	*P* = 1.00; *I*^2^ = 0%	RR (M-H, Fixed, 95% CI)	3.06 [0.49, 19.25]	*P* = 0.23
24 h-mortality (non-RCT)	3	831	Not applicable	RR (M-H, Fixed, 95% CI)	1.23 [0.94, 1.62]	*P* = 0.13
24 h-mortality (CPR)	1	229	Not applicable	RR (M-H, Fixed, 95% CI)	1.23 [0.94, 1.62]	*P* = 0.13
24 h-mortality (non-CPR)	5	1248	*P* = 1.00; *I*^2^ = 0%	RR (M-H, Fixed, 95% CI)	3.06 [0.49, 19.25]	*P* = 0.23
24 h-mortality (experienced)	1	164	Not applicable	RR (M-H, Fixed, 95% CI)	3.07 [0.13, 74.35]	*P* = 0.49
24 h-mortality (inexperienced)	5	1313	*P* = 0.73; *I*^2^ = 0%	RR (M-H, Fixed, 95% CI)	1.27 [0.97, 1.67]	*P* = 0.08
28 d-mortality	7	1821	*P* = 0.98; *I*^2^ = 0%	RR (M-H, Fixed, 95% CI)	1.04 [0.92, 1.19]	*P* = 0.52
28 d-mortality (RCT)	5	1382	*P* = 0.90; *I*^2^ = 0%	RR (M-H, Fixed, 95% CI)	1.04 [0.86, 1.26]	*P* = 0.66
28 d-mortality (non-RCT)	2	439	*P* = 0.82; *I*^2^ = 0%	RR (M-H, Fixed, 95% CI)	1.04 [0.88, 1.23]	*P* = 0.62
28 d-mortality (CPR)	1	229	Not applicable	RR (M-H, Fixed, 95% CI)	1.03 [0.86, 1.23]	*P* = 0.76
28 d-mortality (non-CPR)	6	1592	*P* = 0.95; *I*^2^ = 0%	RR (M-H, Fixed, 95% CI)	1.05 [0.89, 1.24]	*P* = 0.57
28 d-mortality (experienced)	2	821	*P* = 0.94; *I*^2^ = 0%	RR (M-H, Fixed, 95% CI)	1.22 [0.83, 1.79]	*P* = 0.32
28 d-mortality (inexperienced)	5	1000	*P* = 0.99; *I*^2^ = 0%	RR (M-H, Fixed, 95% CI)	1.01 [0.88, 1.16]	*P* = 0.88

Abbreviations: *EI* Esophageal intubation, *CA* Cardiac arrest, *RCT* Randomized controlled trial, *CPR* Cardiopulmonary resuscitation, *RR* Risk ratio, *M-H* Mantel-Haenszel

Three studies reported the incidence of hypoxemia [9, 14, 19]. One study, which reported the rate of decline in oxygen saturation greater than 10% from baseline [5], was also included in the pooled analysis. All of them are non-CPR studies. Eight studies reported the incidence of severe hypoxemia [4, 6, 7, 9, 14, 17, 19, 20], all of them are non-CPR studies. Thirteen studies reported the incidence of aspiration [4, 5, 7, 9, 13, 14, 17, 19–22, 39, 40]. Seven studies reported the incidence of new-onset cardiac arrest [4, 5, 12, 14, 17, 19, 20]. Six studies reported short-term mortality within 24 h [4, 7, 8, 14, 19, 20], and 7 studies reported long-term mortality (28 d or in-hospital) [4, 8, 13, 16, 17, 19, 22]; among them, one reported the data based on the number of intubations rather than the number of participants [4]. The available data was used because both the number of intubations and that of participants were quite similar. Pooled analyses for all these outcomes showed no significant differences between VL and DL (*P > 0.05*). For these adverse events, however, there was no significant heterogeneity among studies (*I*^2^ < 40%). Subgroup analyses for all these adverse events based on the type of studies, whether a CPR study, or operators’ expertise showed no significant difference between VL and DL in all subgroups (*P > 0.05*) except for the incidence of hypoxemia when intubated by inexperienced operators (*P* = 0.03).

The incidences of hypoxemia and severe hypoxemia in both groups were almost 2–3 times higher in the non-RCTs than in the RCTs (21–30% vs. 11–12% for hypoxemia; 10–12% vs. 6–6.7% for severe hypoxemia, *P* > 0.05). The incidence of aspiration in both groups was higher in the non-RCTs than in the RCTs (3–4.5% vs. 2.5–2.7%, *P* > 0.05).

#### Trial sequential analysis

The TSA of a diversity-adjusted required information size for the rate of EI was 668 patients. The cumulative z-curve crossed the boundary of required information size and TSA monitoring boundary for favoring VL. Thus, this pooled analysis from RCTs is conclusive, namely, the use of VL reduces the rate of EI. For incidences of aspiration and new-onset cardiac arrest, and short-term mortality, the cumulative z-curve did not cross the boundary of required information size and TSA monitoring boundary. Thus, the pooled analyses from RCTs for these adverse outcomes are inconclusive. For incidence of hypoxemia and long-term mortality, boundary TSA is ignored due to too little information size. Thus, the pooled analyses from RCTs for these adverse outcomes are also inconclusive (Additional file 1: Fig. S20A-G and Additional file 2: Table S2).

In summary, pooled analysis showed that the use of VL reduced the rate of EI, especially for inexperienced operators. As to the other adverse outcomes, however, no significant differences were identified, except for the incidence of hypoxemia when intubated by inexperienced operators.

## Discussion

This systematic review and meta-analysis is focused on adverse events of tracheal intubation using VL compared with DL in the ED and ICU patients by including both RCTs and observational studies. Pooled analysis showed that the use of VL reduced the rate of EI; as to the other adverse outcomes, however, no significant differences were identified.

The rate of EI was chosen as the primary outcome in this analysis, as even a single episode of recognized EI is significantly associated with desaturation, increased risk of aspiration and cardiac arrest. It has been shown that patients with EI have higher incidences of aspiration (6.1 times), dysrhythmia (6.4 times), hypotension (3.1 times), and hypoxemia [3]. In our analysis, however, a significant lower rate of EI by using VL did not result in any significant difference for other adverse events. This is probably due to small sample size, leading these results inconclusive, as the TSA has proved. Even though, a lower trend for the incidences of hypoxemia and aspiration with VL can still be identified. Inexperienced operators benefited more from using VL, with significant lower incidences of EI and hypoxemia. For inexperienced operators who have not performed the tracheal intubation with VL and DL, visualization of the airway on VL screen can allow their supervisors to directly assist them in completing tracheal intubation themselves, thus improving the success rate [42]. In contrast, experienced operators with extensive training and experience on the tracheal intubation using DL might overshadow the benefits of VL, especially when patients have a normal airway or there are some VL-related difficult scenarios like secretions or blood in the airway or certain VL design-related deficiencies which may bate their benefits [19, 22, 43, 44].

Different from above non-fatal adverse outcomes, an increased trend was otherwise shown for incidence of new-onset cardiac arrest and short-term mortality by using VL. However, it should be noted that among the studies reporting the results of new-onset cardiac arrest and short-term mortality, only one was the study in which  emergency tracheal  intubation was performed by experienced operators [14]. The longer duration of intubation with VL by inexperienced operators may result in an increased risk of severe life-threatening adverse outcomes [19]. In addition, the pooled result of the short-term mortality was mainly from a CPR study [8]. Although the participants were randomized, the baseline clinical characteristics for both groups was not comparable, with more ischemic heart diseases in the VL group, leading the patients in this group to be at a higher risk of severe adverse outcomes. It has been reported that the use of a VL can reduce the chest compression interruptions for both experienced and inexperienced operators [11, 18]. However, visualization of VL can be unfavorably compromised by increased amount of secretions and emesis in the upper airway, which are common during the CPR, leading to a longer duration of intubation which may worsen the patient’s prognosis [16, 22]. Besides traditional airway suctioning, other techniques to decrease influences of massive secretions and emesis on airway visualization and intubation procedure, such as intentional esophageal intubation (IEI) [45] and suction-assisted laryngoscopy and airway decontamination (SALAD) [46], have been described. Furthermore, the use of airway decontamination technique provides improved intubation conditions with a VL. However, these methods appear mostly in case report or simulated mannequin study and have not been generalized in clinical practice. Nowadays, even for skilled operators like trained anesthesiologists, DL might still be the first choice for urgent tracheal intubation during the CPR, especially in patients without difficult airways.

Our study included RCTs and observational studies. The overall risk assessment of bias for the included RCTs was classified as low risk. Although blinding was not adopted in the most RCTs, we judged “no blinding” as low risk, as it seems impossible to blind personnel in the urgent situations at times. The overall risk assessment of bias for the included observational studies was classified as moderate or severe risk, mainly due to the confounding factors. For most outcomes, the level of heterogeneity was low, but subgroup and sensitivity analyses based on some potential clinical heterogeneous factors had been performed in our analysis. Furthermore, the TSA ensured the credibility of the result of primary endpoint from RCTs.

There are some limitations in our analysis that deserve special attentions. First and foremost, the inclusion of observational studies inevitably introduces selection bias, leading the possibility that patients in the VL and DL groups differ significantly in terms of operator’s expertise, anesthesia methods, certain difficult airway characteristics, clinical scenarios, and even basic characteristics of patients [4–6, 8–10]. Besides the disparities between groups, a reporting bias might be also present due to self-reported or recall property of the data in the observational studies, leading to the inaccuracy of data collection. Second, some of the included studies were quality-improvement process within a before–after study [4, 9]. The better result may probably not only from the switch from DL to VL, but also from the improved patient management in quality-improvement studies. Third, during the searching process, 12 observational studies were found to be carried out in a same ED or ICU from a same hospital with an overlapped study period [3, 5, 10, 47–55]. Only the latest 3 large studies [3, 5, 10] covering the majority of other 8 studies were decided to be included. This might lose some validated participants while avoiding repeated enrolment. Fourth, definition of the expertise used in our analysis was somewhat arbitrary. A Cochrane review defines an experienced operator as a clinician with more than 20 tracheal intubations with each device and thence obtains a fewer failed intubations when using VL [56]. However, it is suggested that the number of procedures required to achieve proficiency with tracheal intubation using DL in an controlled environment like OR is approximately 50 [57, 58]. Given the challenges of limited cardiopulmonary reserve, difficulty on airway assessment, few chances of tracheal intubation practice in the ED or ICU, it is even hard to know how many procedures are required to achieve a definitive competence with tracheal intubation in this patient population. Anyhow, having one VL available for all urgent tracheal intubation could potentially offer a major safety advantage. Fifth, there might be still some other heterogeneous factors in our analysis, such as different types of VLs used and different in-hospital setting, i.e. ED, ICU, or general ward. About one third of our included studies used more than one type of VL [3–5, 8, 10, 17, 19], making subgroup analysis on this factor difficult. Although ED and ICU are two main in-hospital settings for urgent tracheal intubation, indications, intubation conditions, and severity of patients’ illness would not differ significantly, at least not as much as the difference between pre-hospital and in-hospital setting. Lastly, some studies used the episode of intubations or attempts instead of participants [3, 4, 12]. When calculating incidence of adverse outcomes, this would be inappropriate. However, sensitivity analysis by excluding these studies did not change the result of primary endpoint.

## Conclusions

This systematic review and meta-analysis reveals that VL can reduce the risk of EI during urgent tracheal intubations in the ED and ICU patients, but does not provide significant benefits on other adverse events associated with tracheal intubation. Further studies are needed to demonstrate whether severe adverse events like cardiac arrest or mortality are significantly different between two devices. Furthermore, well-designed RCTs are further needed to focus on a specific scenario and should stratify some other prognostic indicators such as length of hospital stay and cost.

## Supplementary information

**Additional file 1: Fig. S1-S20.** The funnel plot obtained from primary outcome. **Fig. S2**. Forest plot for comparison of rate of esophageal intubation between video laryngoscope (VL) and direct laryngoscope (DL). M-H, Mantel–Haenszel. **Fig. S3.** Forest plot for comparison of rate of esophageal intubation based on whether a CPR study between video laryngoscope (VL) and direct laryngoscope (DL). M-H, Mantel–Haenszel. **Fig. S4.** Forest plot for comparison of rate of esophageal intubation based on experience of operators between video laryngoscope (VL) and direct laryngoscope (DL). M-H, Mantel–Haenszel. **Fig. S5.** Forest plot for comparison of incidence of hypoxemia based on the type of studies between video laryngoscope (VL) and direct laryngoscope (DL). M-H, Mantel–Haenszel. **Fig. S6.** Forest plot for comparison of incidence of hypoxemia based on experience of operators between video laryngoscope (VL) and direct laryngoscope (DL). M-H, Mantel–Haenszel. **Fig. S7.** Forest plot for comparison of incidence of severe hypoxemia based on the type of studies between video laryngoscope (VL) and direct laryngoscope (DL). M-H, Mantel–Haenszel. **Fig. S8.** Forest plot for comparison of incidence of severe hypoxemia based on experience of operators between video laryngoscope (VL) and direct laryngoscope (DL). M-H, Mantel–Haenszel. **Fig. S9.** Forest plot for comparison of incidence of aspiration based on the type of studies between video laryngoscope (VL) and direct laryngoscope (DL). M-H, Mantel–Haenszel. **Fig. S10.** Forest plot for comparison of incidence of aspiration based on whether a CPR study between video laryngoscope (VL) and direct laryngoscope (DL). M-H, Mantel–Haenszel. **Fig. S11.** Forest plot for comparison of incidence of aspiration based on experience of operators between video laryngoscope (VL) and direct laryngoscope (DL). M-H, Mantel–Haenszel. **Fig. S12.** Forest plot for comparison of incidence of new onset of cardiac arrest based on the type of studies between video laryngoscope (VL) and direct laryngoscope (DL). M-H, Mantel–Haenszel. **Fig. S13.** Forest plot for comparison of incidence of new onset of cardiac arrest based on experience of operators between video laryngoscope (VL) and direct laryngoscope (DL). M-H, Mantel–Haenszel. **Fig. S14.** Forest plot for comparison of 24 h-mortality based on the type of studies between video laryngoscope (VL) and direct laryngoscope (DL). M-H, Mantel–Haenszel. **Fig. S15.** Forest plot for comparison of 24 h-mortality based on whether a CPR study between video laryngoscope (VL) and direct laryngoscope (DL). M-H, Mantel–Haenszel. **Fig. S16.** Forest plot for comparison of 24 h-mortality based on experience of operators between video laryngoscope (VL) and direct laryngoscope (DL). M-H, Mantel–Haenszel. **Fig. S17.** Forest plot for comparison of 28d-mortality based on the type of studies between video laryngoscope (VL) and direct laryngoscope (DL). M-H, Mantel–Haenszel. **Fig. S18.** Forest plot for comparison of 28 d-mortality based on whether a CPR study between video laryngoscope (VL) and direct laryngoscope (DL). M-H, Mantel–Haenszel. **Fig. S19.** Forest plot for comparison of 28 d-mortality based on experience of operators between video laryngoscope (VL) and direct laryngoscope (DL). M-H, Mantel–Haenszel. **Fig. S20.** The TSA for (a) rate of esophageal intubation, (b) incidence of hypoxemia, (c) incidence of severe hypoxemia, (d) incidence of aspiration, (e) incidence of new-onset cardiac arrest, (f) 24 h-mortality, (g) 28 d-mortality based on 5% risk of type-1 error (one-sided upper), power 80%, low bias-based relative risk reduction and incidence in control arm with a model variance-based heterogeneity correction.

**Additional file 2: Tables S1-S2.** The GRADE for all adverse events. The TSA for all adverse events from randomized controlled trials.

## Data Availability

All data generated or analyzed during this study are included in this published article.
