# Supplementary material for: Comparison of adverse events between video and direct laryngoscopes for tracheal intubations in emergency department and ICU patients–a systematic review and meta-analysis
Source: Scand J Trauma Resusc Emerg Med. 2020 Feb 7;28:10. doi: 10.1186/s13049-020-0702-7 (PMC7006069; doi:10.1186/s13049-020-0702-7)
Supplement: Supplementary file 1 — Additional file 1: Fig. S1-S20. The funnel plot obtained from primary outcome. Fig. S2. Forest plot for comparison of rate of esophageal intubation between video laryngoscope (VL) and direct laryngoscope (DL). M-H, Mantel–Haenszel. Fig. S3. Forest plot for comparison of rate of esophageal intubation based on whether a CPR study between video laryngoscope (VL) and direct laryngoscope (DL). M-H, Mantel–Haenszel. Fig. S4. Forest plot for comparison of rate of esophageal intubation based on experience of operators between video laryngoscope (VL) and direct laryngoscope (DL). M-H, Mantel–Haenszel. Fig. S5. Forest plot for comparison of incidence of hypoxemia based on the type of studies between video laryngoscope (VL) and direct laryngoscope (DL). M-H, Mantel–Haenszel. Fig. S6. Forest plot for comparison of incidence of hypoxemia based on experience of operators between video laryngoscope (VL) and direct laryngoscope (DL). M-H, Mantel–Haenszel. Fig. S7. Forest plot for comparison of incidence of severe hypoxemia based on the type of studies between video laryngoscope (VL) and direct laryngoscope (DL). M-H, Mantel–Haenszel. Fig. S8. Forest plot for comparison of incidence of severe hypoxemia based on experience of operators between video laryngoscope (VL) and direct laryngoscope (DL). M-H, Mantel–Haenszel. Fig. S9. Forest plot for comparison of incidence of aspiration based on the type of studies between video laryngoscope (VL) and direct laryngoscope (DL). M-H, Mantel–Haenszel. Fig. S10. Forest plot for comparison of incidence of aspiration based on whether a CPR study between video laryngoscope (VL) and direct laryngoscope (DL). M-H, Mantel–Haenszel. Fig. S11. Forest plot for comparison of incidence of aspiration based on experience of operators between video laryngoscope (VL) and direct laryngoscope (DL). M-H, Mantel–Haenszel. Fig. S12. Forest plot for comparison of incidence of new onset of cardiac arrest based on the type of studies between video laryngoscope (VL) an [file 13049_2020_702_MOESM1_ESM.docx]

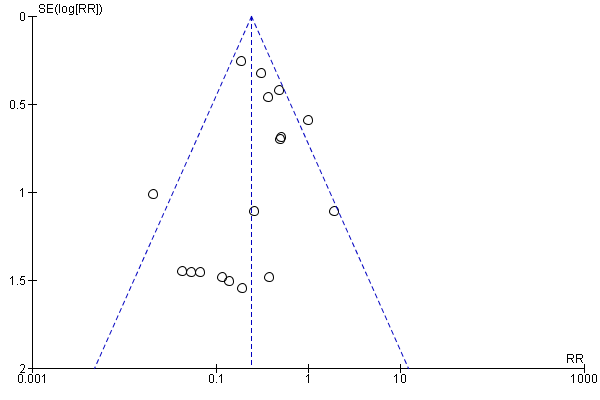


**Fig. S1.** The funnel plot obtained from primary outcome


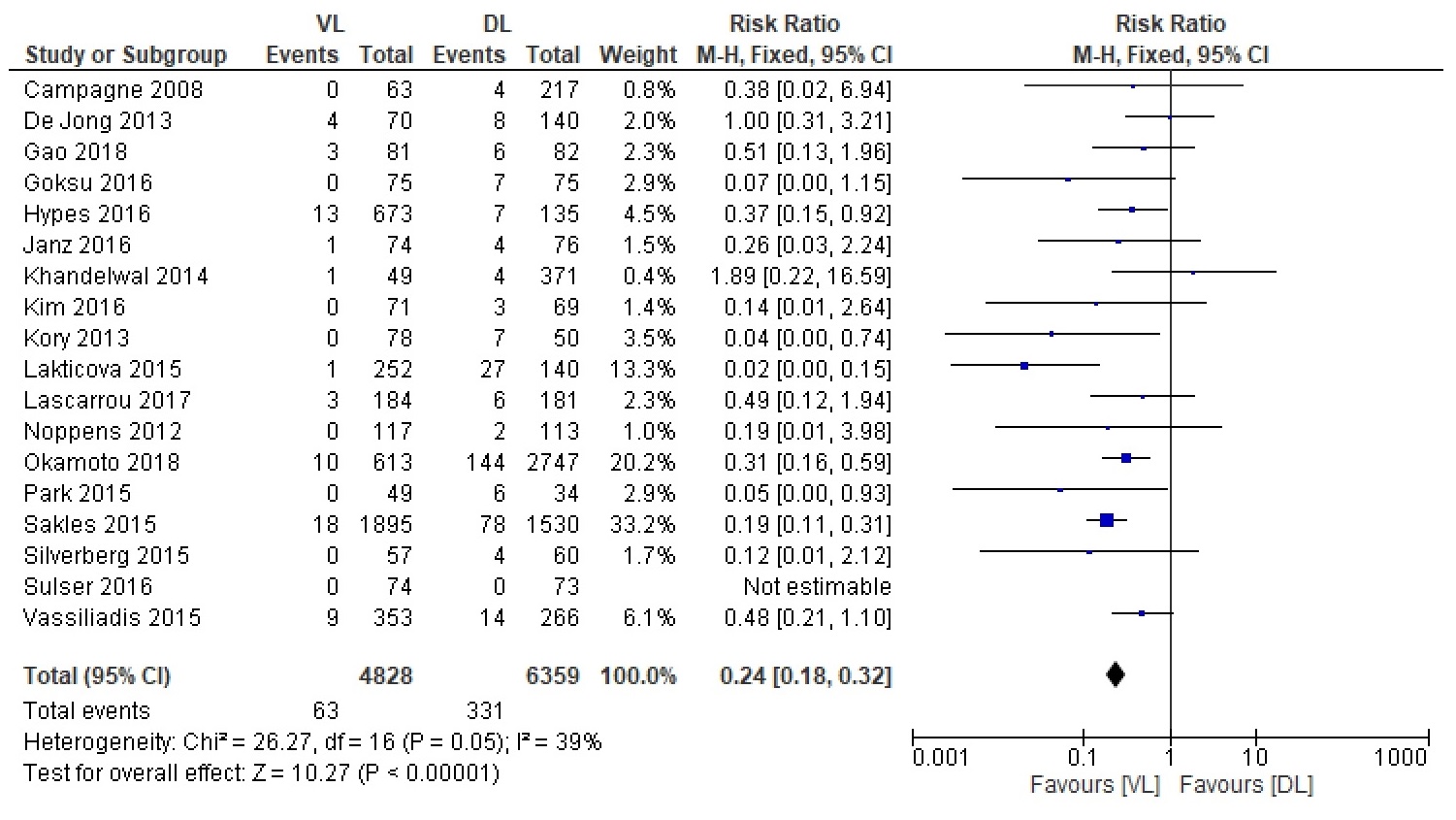


**Fig. S2** Forest plot for comparison of rate of esophageal intubation between video laryngoscope (VL) and direct laryngoscope (DL). M-H, Mantel–Haenszel.


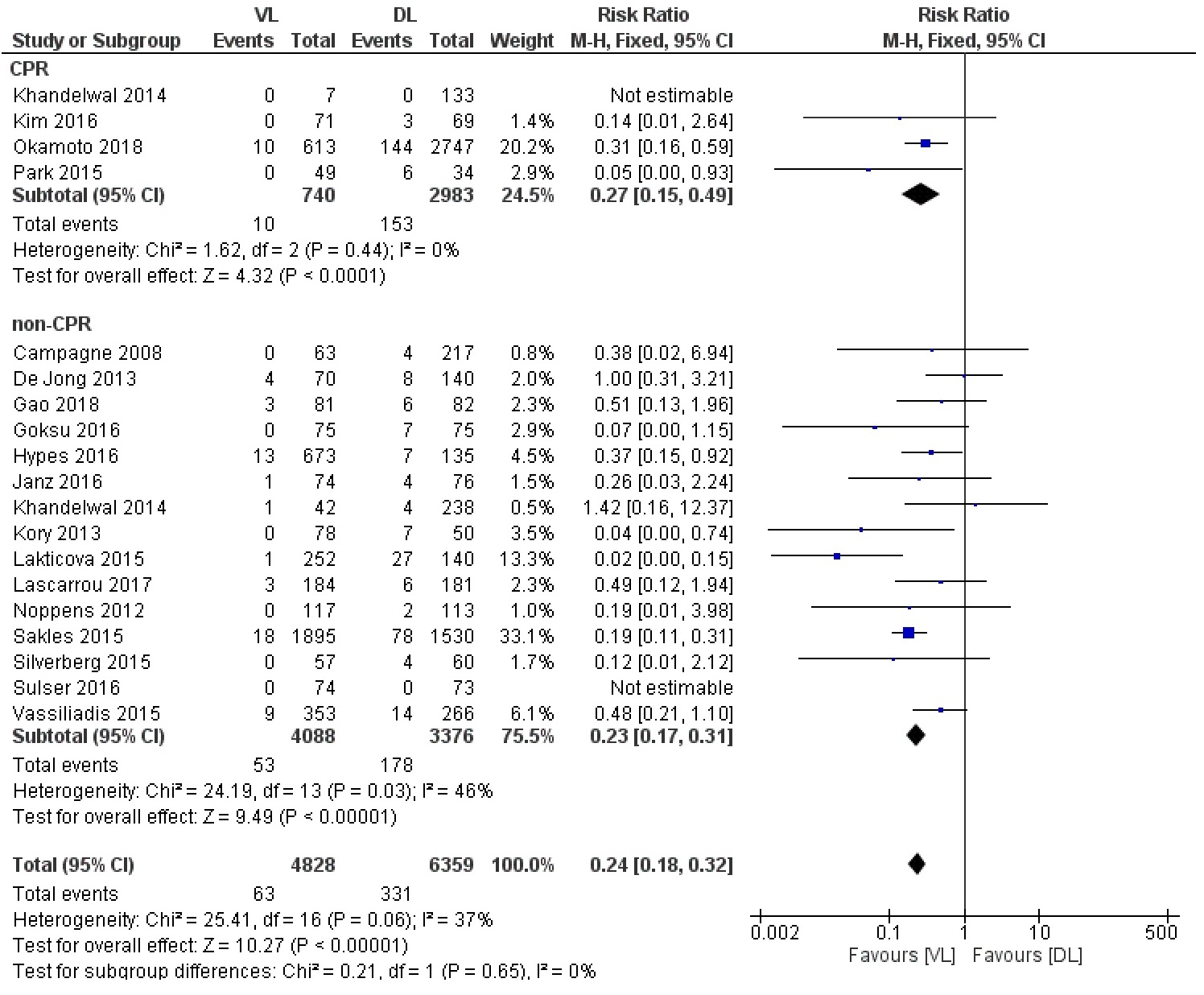


**Fig. S3** Forest plot for comparison of rate of esophageal intubation based on whether a CPR study between video laryngoscope (VL) and direct laryngoscope (DL). M-H, Mantel-Haenszel.


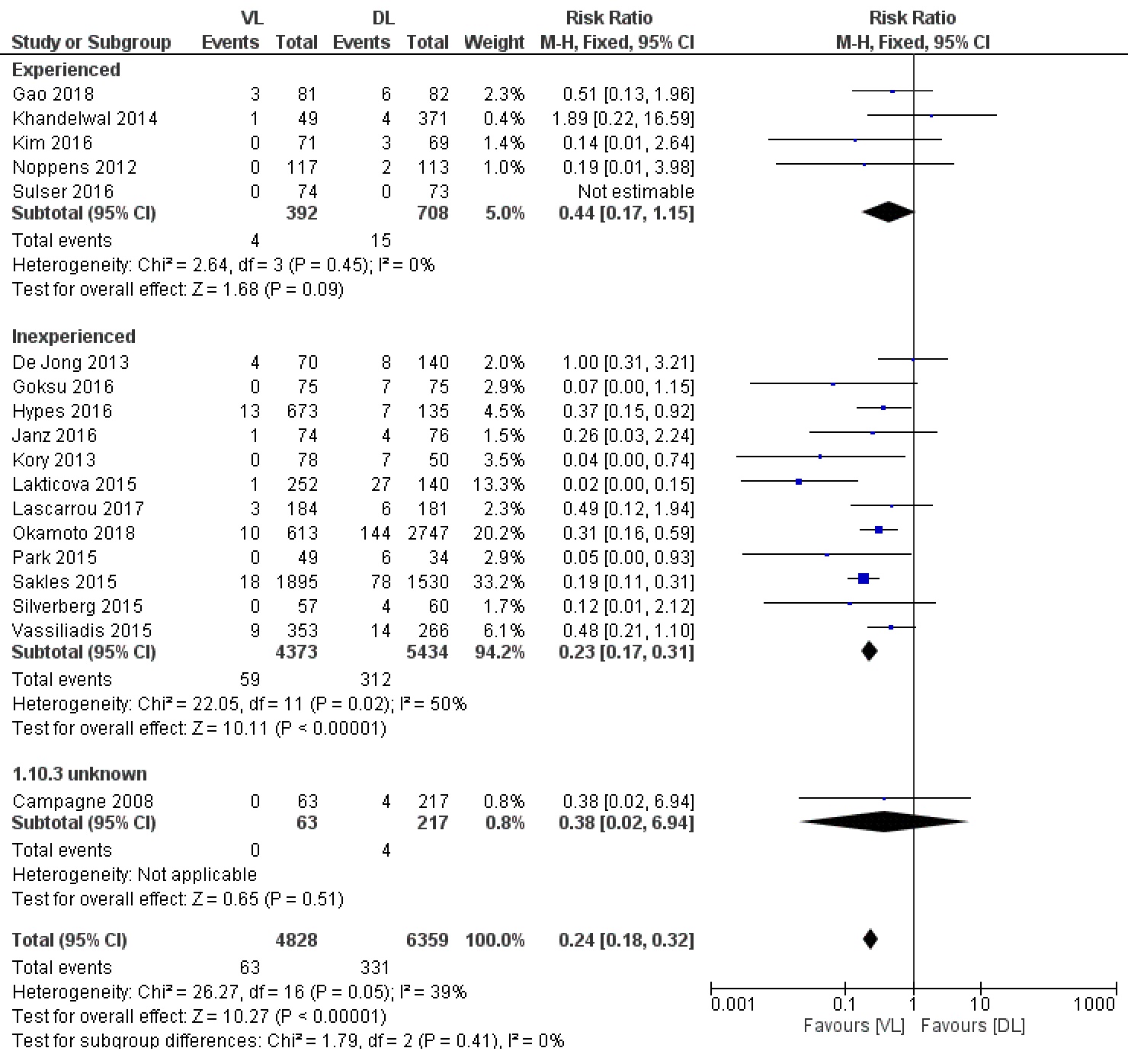


**Fig. S4** Forest plot for comparison of rate of esophageal intubation based on the experience of the operators between video laryngoscope (VL) and direct laryngoscope (DL). M-H, Mantel-Haenszel.


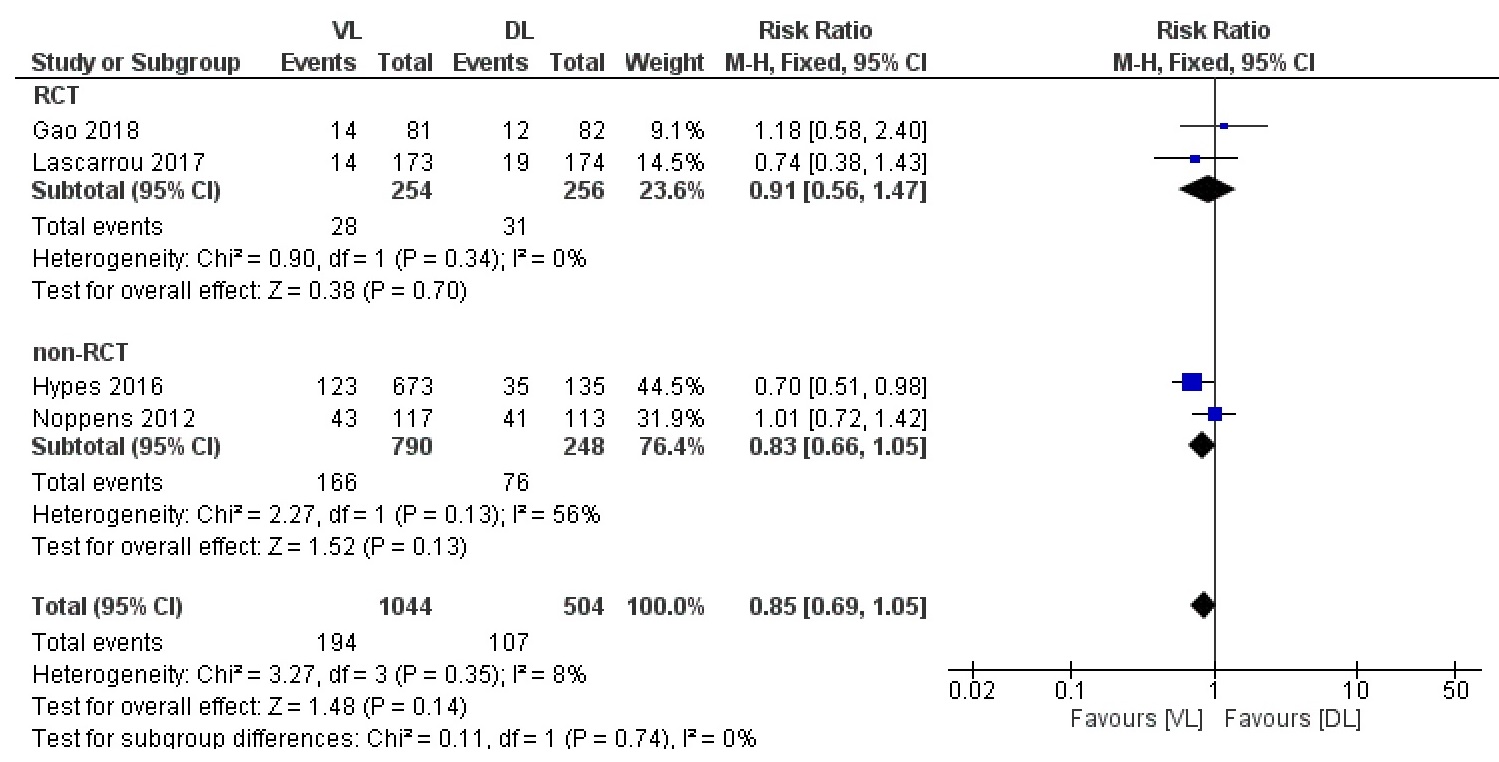


**Fig. S5** Forest plot for comparison of incidence of hypoxemia based on the type of studies between video laryngoscope (VL) and direct laryngoscope (DL). M-H, Mantel-Haenszel.


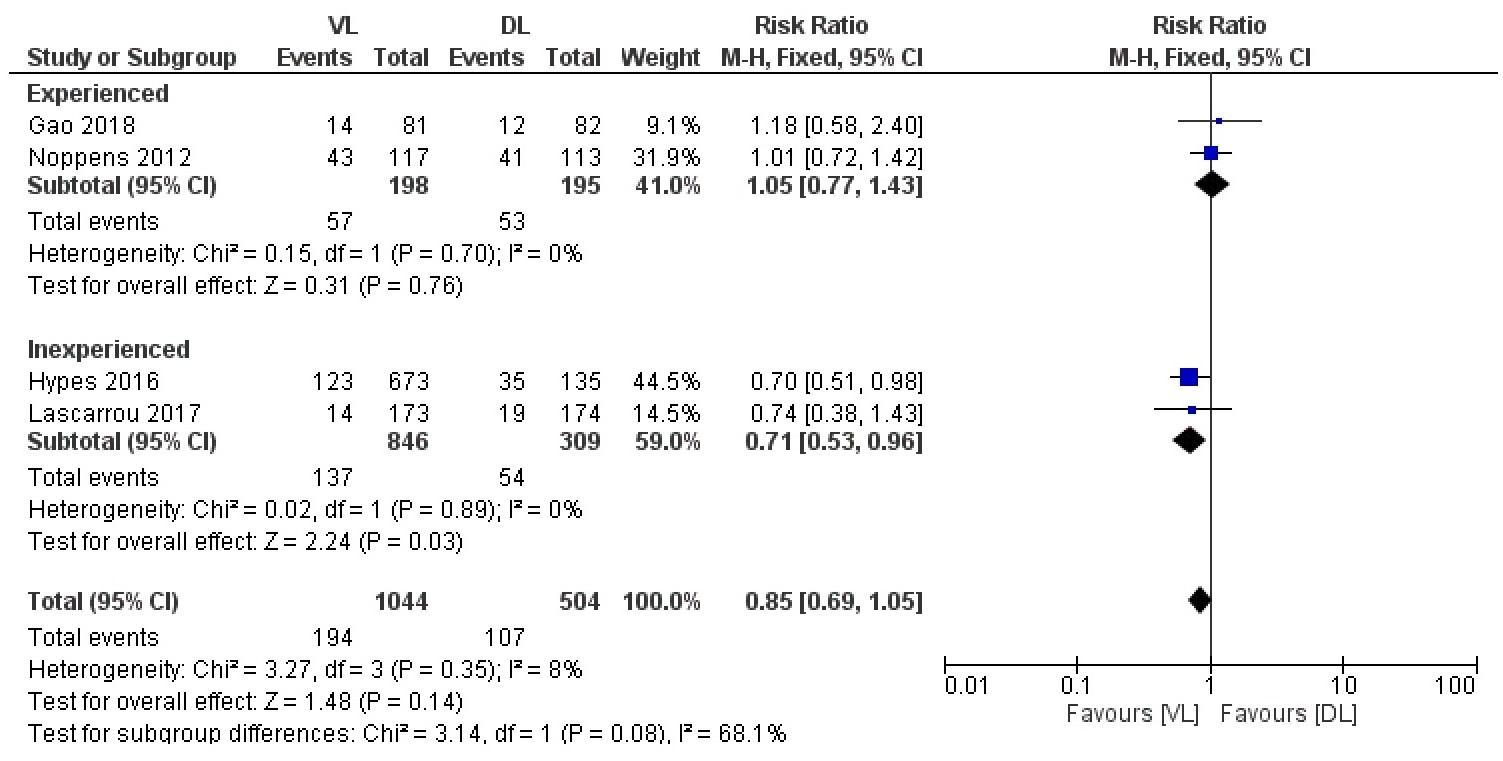


**Fig. S6** Forest plot for comparison of incidence of hypoxemia based on the experience of the operators between video laryngoscope (VL) and direct laryngoscope (DL). M-H, Mantel-Haenszel.


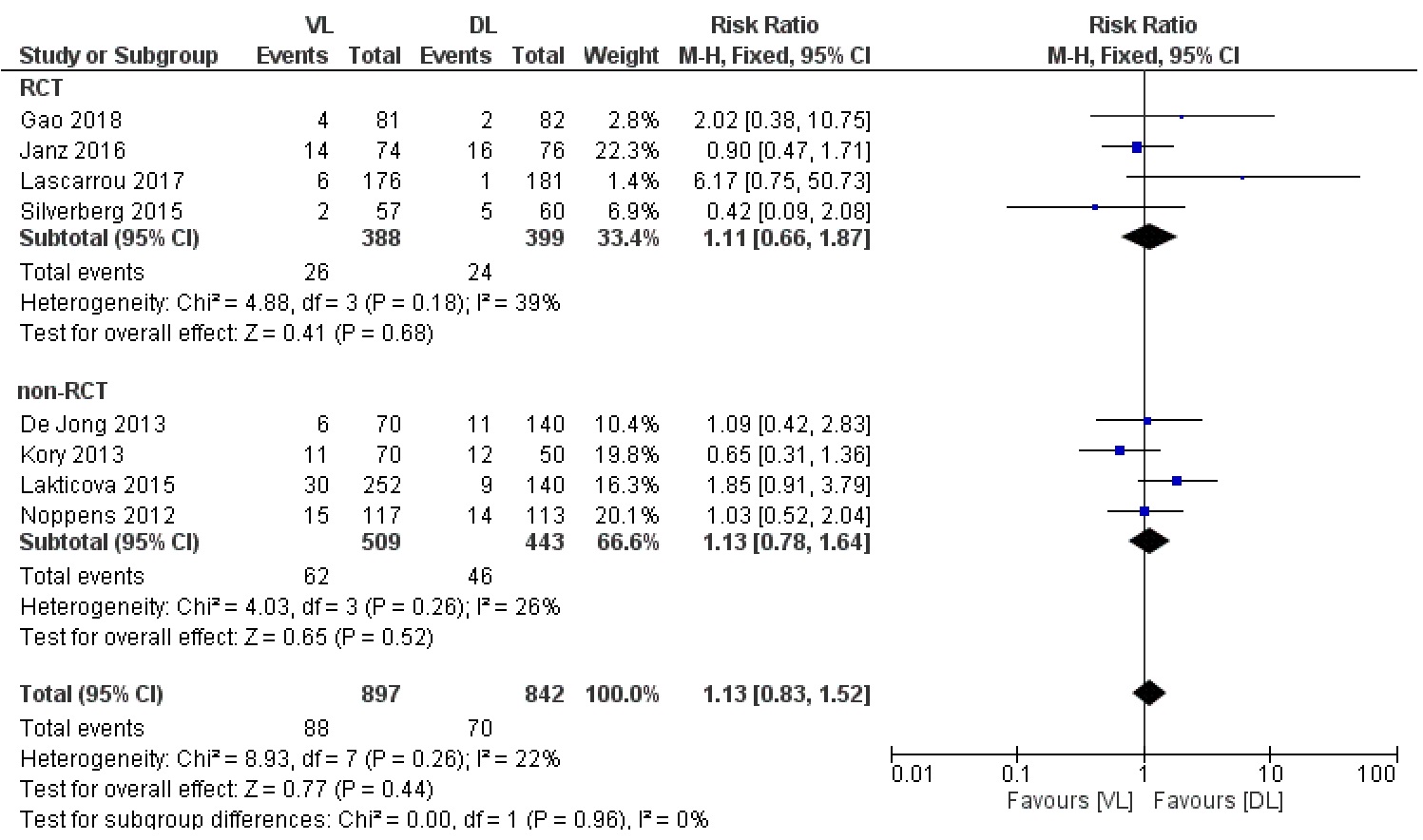


**Fig. S7** Forest plot for comparison of incidence of severe hypoxemia based on the type of studies between video laryngoscope (VL) and direct laryngoscope (DL). M-H, Mantel-Haenszel.


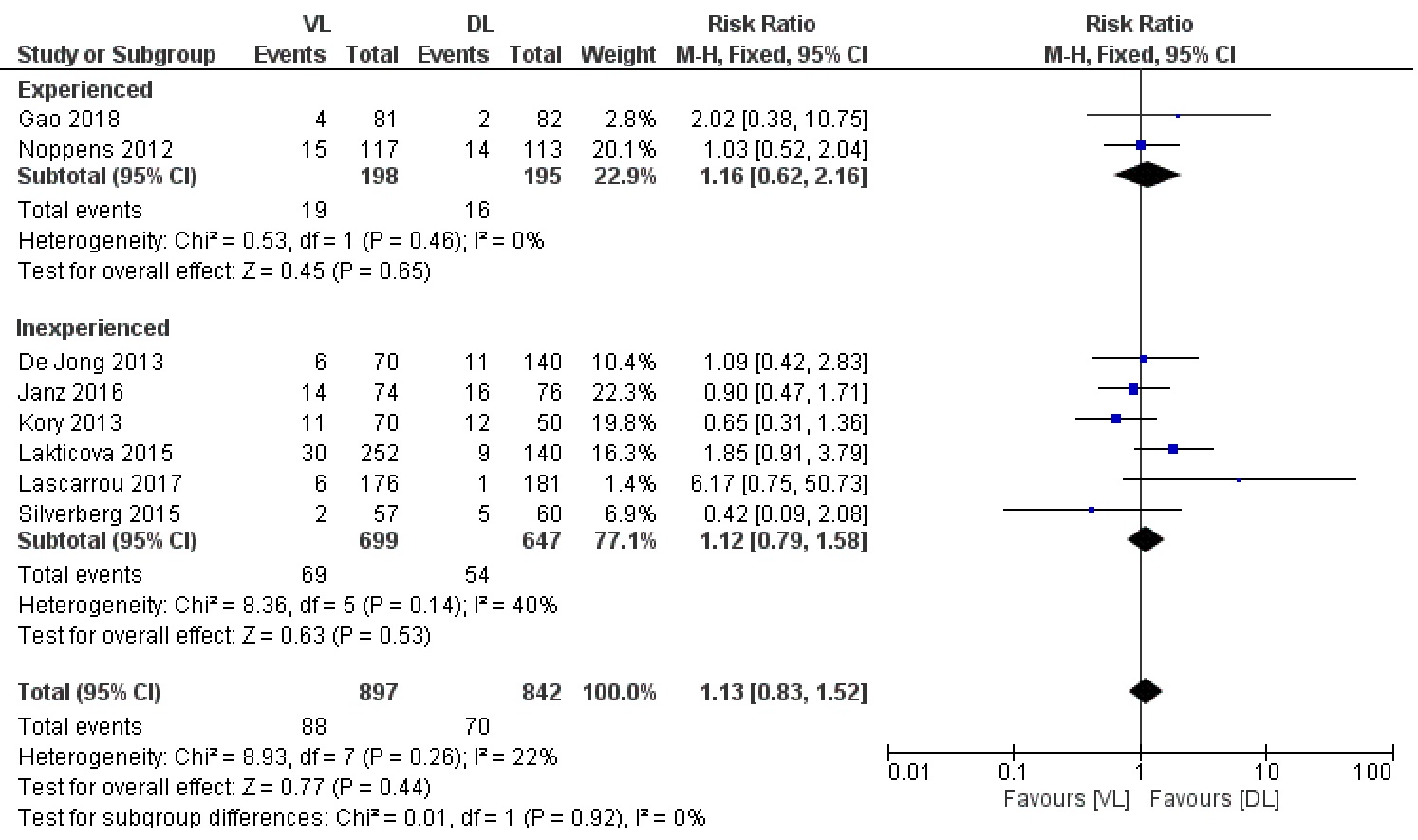


**Fig. S8** Forest plot for comparison of incidence of severe hypoxemia based on the experience of the operators between video laryngoscope (VL) and direct laryngoscope (DL). M-H, Mantel-Haenszel.


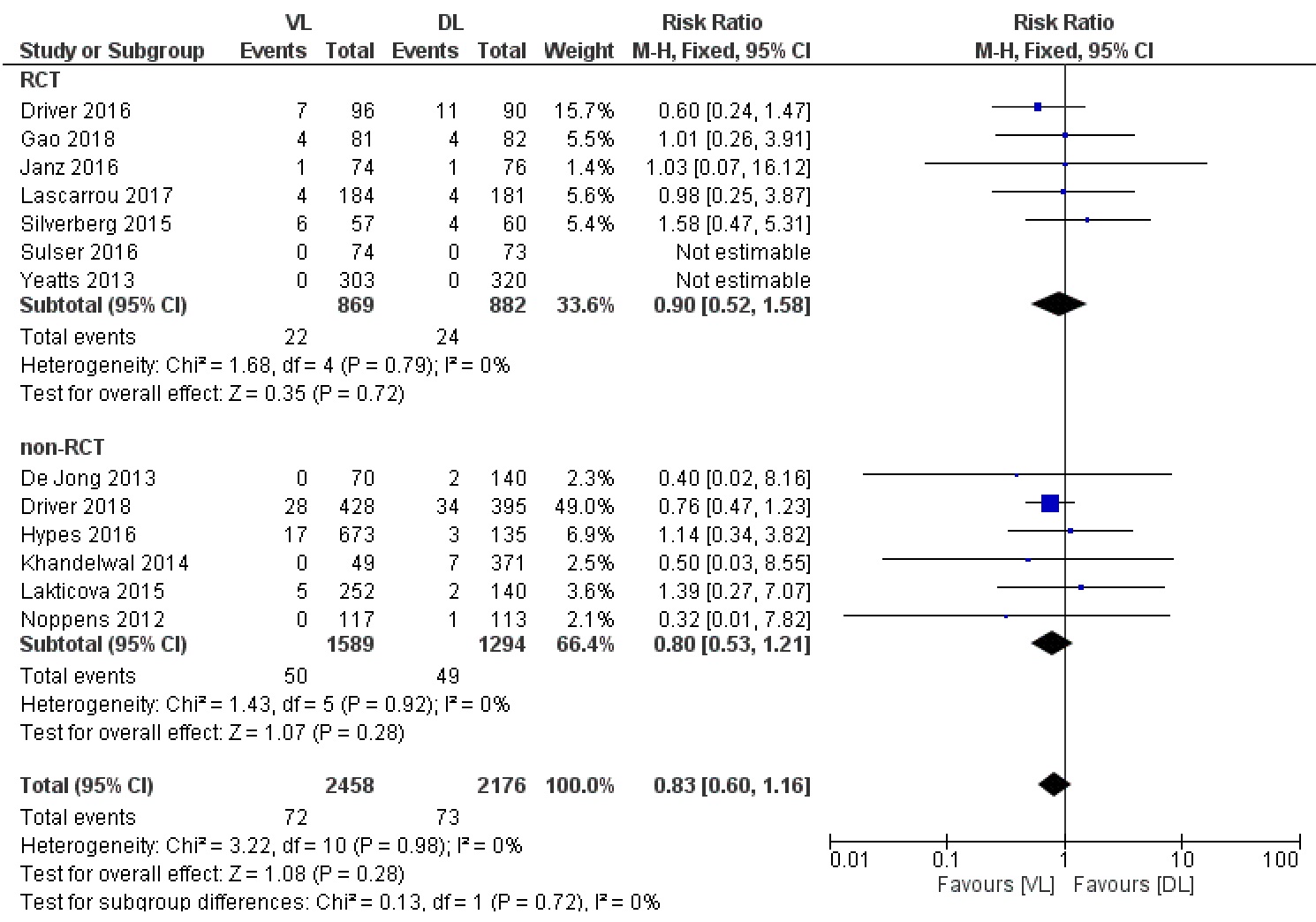


**Fig. S9** Forest plot for comparison of incidence of aspiration based on the type of studies between video laryngoscope (VL) and direct laryngoscope (DL). M-H, Mantel-Haenszel.


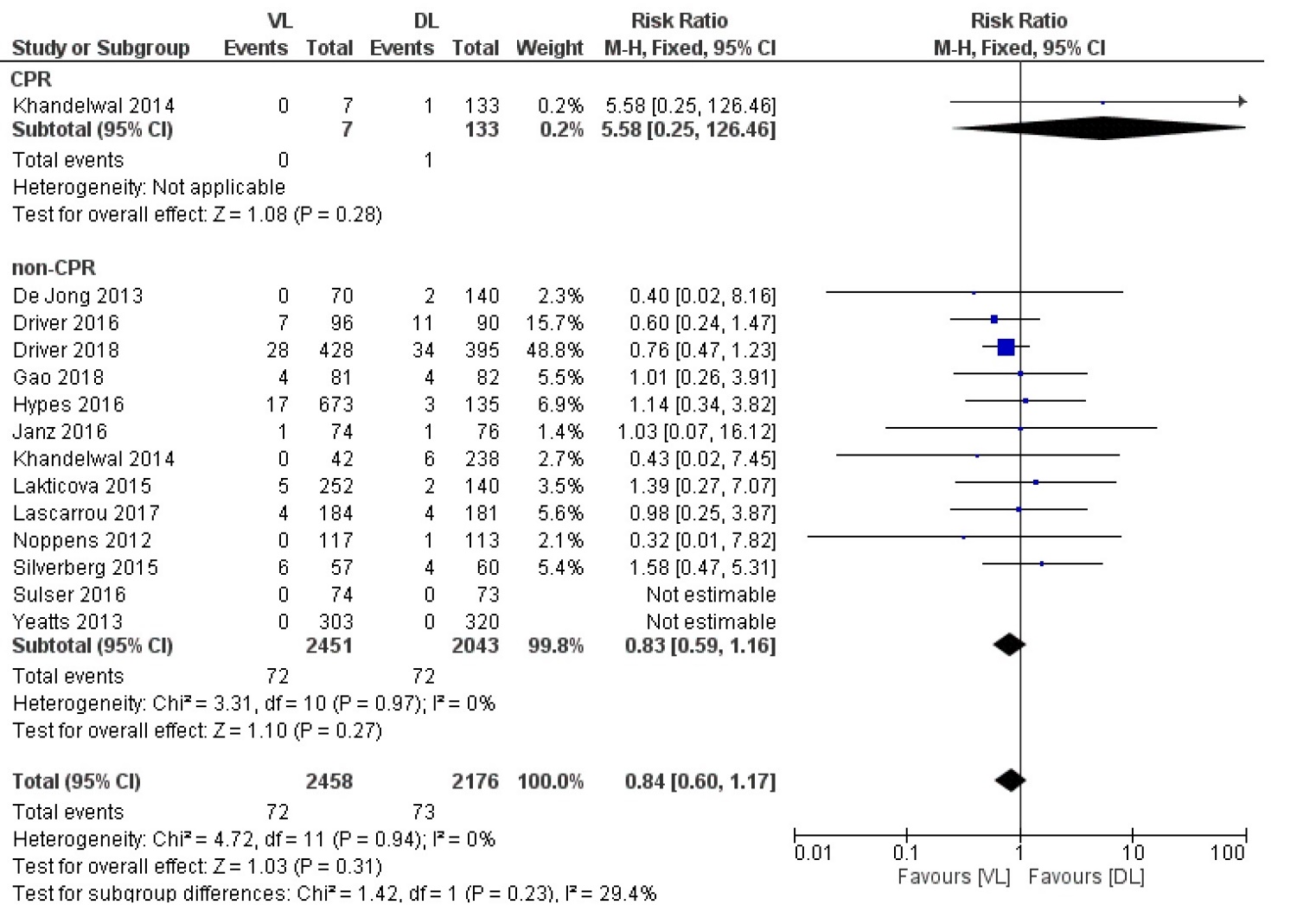


**Fig. S10** Forest plot for comparison of incidence of aspiration based on whether a CPR study between video laryngoscope (VL) and direct laryngoscope (DL). M-H, Mantel-Haenszel.


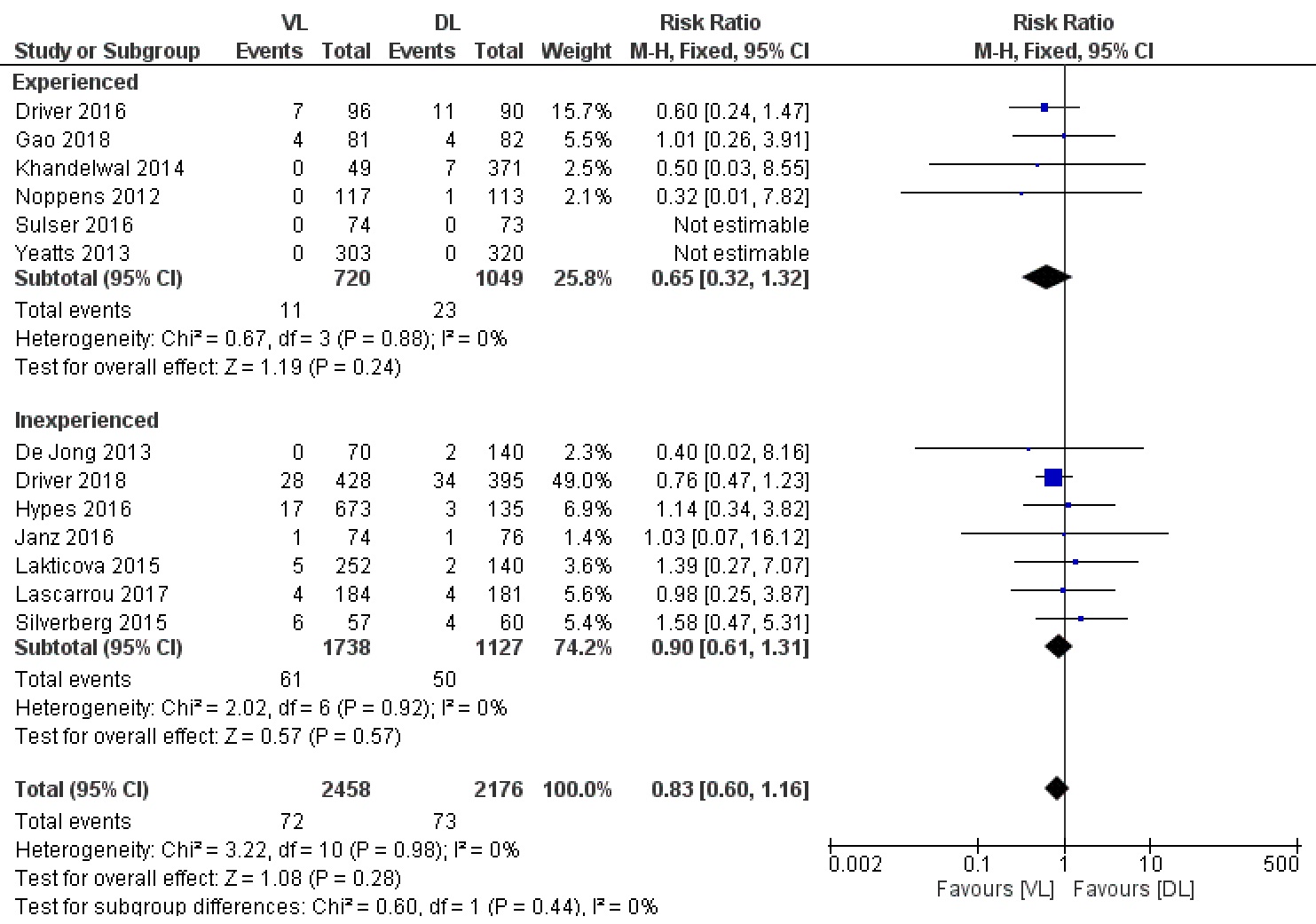


**Fig. S11** Forest plot for comparison of incidence of aspiration based on the experience of the operators between video laryngoscope (VL) and direct laryngoscope (DL). M-H, Mantel-Haenszel.


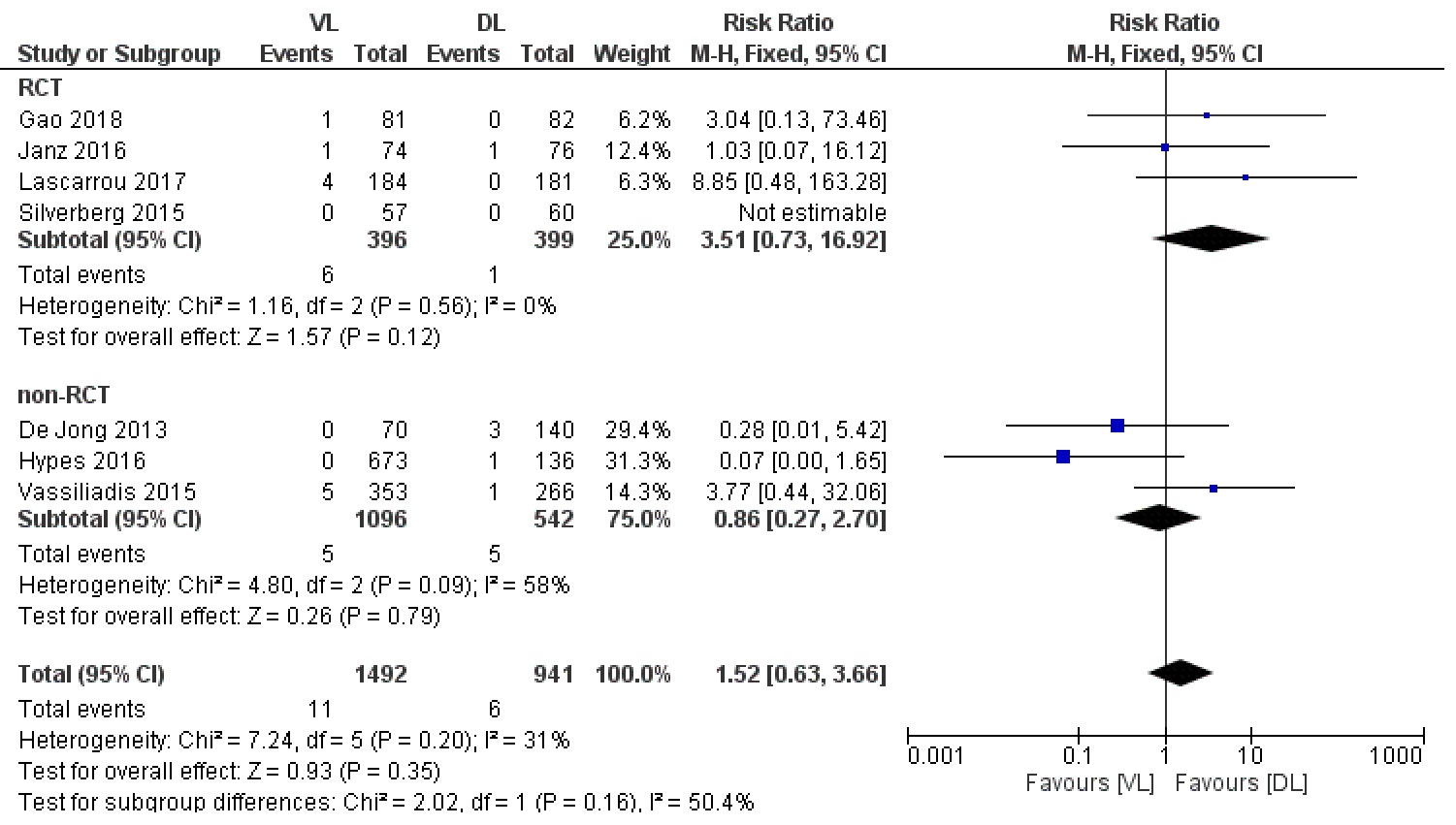


**Fig. S12** Forest plot for comparison of incidence of new onset of cardiac arrest based on the type of studies between video laryngoscope (VL) and direct laryngoscope (DL). M-H, Mantel-Haenszel.


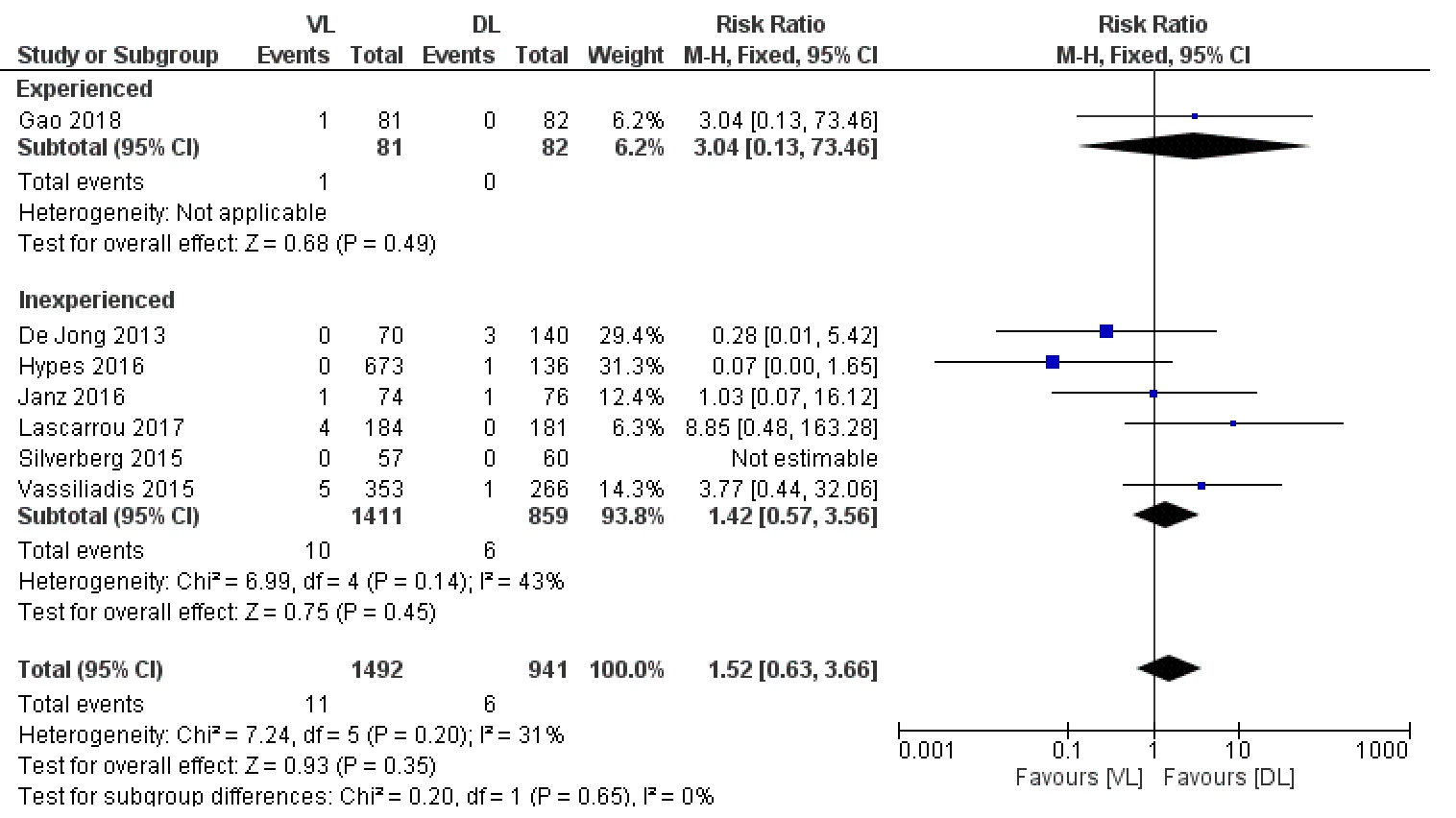


**Fig. S13** Forest plot for comparison of incidence of new onset of cardiac arrest based on the experience of the operators between video laryngoscope (VL) and direct laryngoscope (DL). M-H, Mantel-Haenszel.


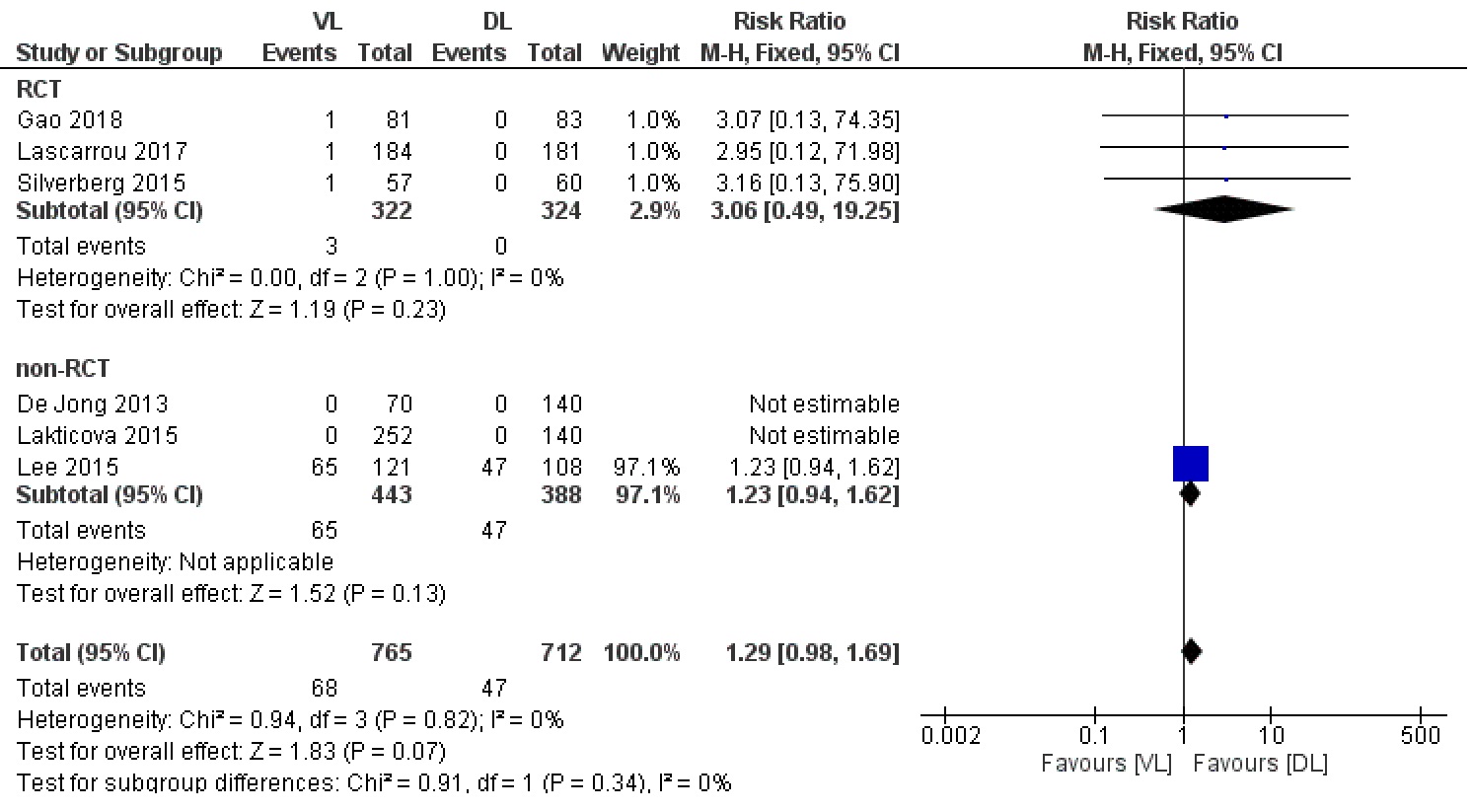


**Fig. S14** Forest plot for comparison of 24 h-mortality based on the type of studies between video laryngoscope (VL) and direct laryngoscope (DL). M-H, Mantel-Haenszel.


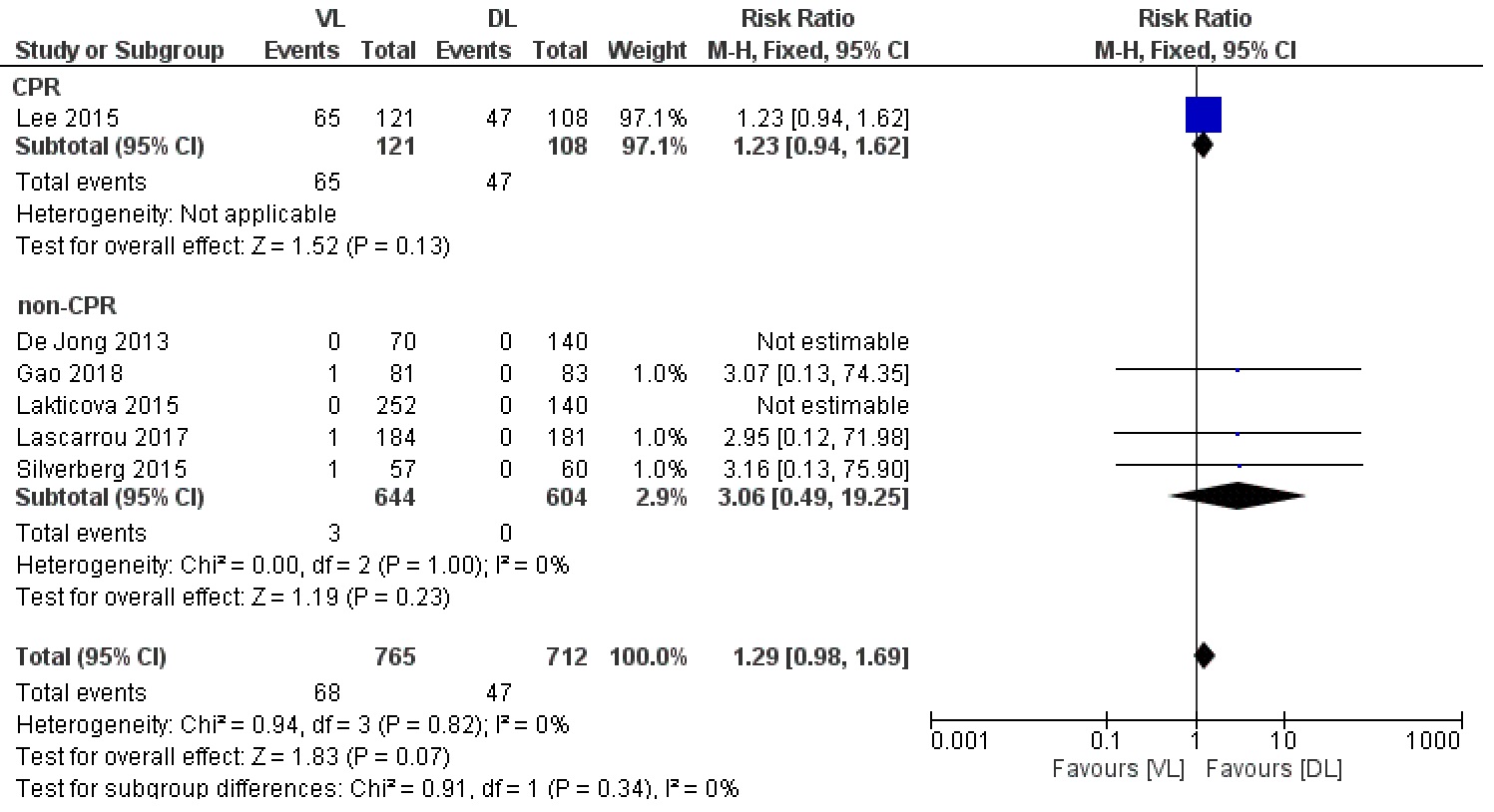


**Fig. S15** Forest plot for comparison of 24h-mortality based on whether a CPR study between video laryngoscope (VL) and direct laryngoscope (DL). M-H, Mantel-Haenszel.


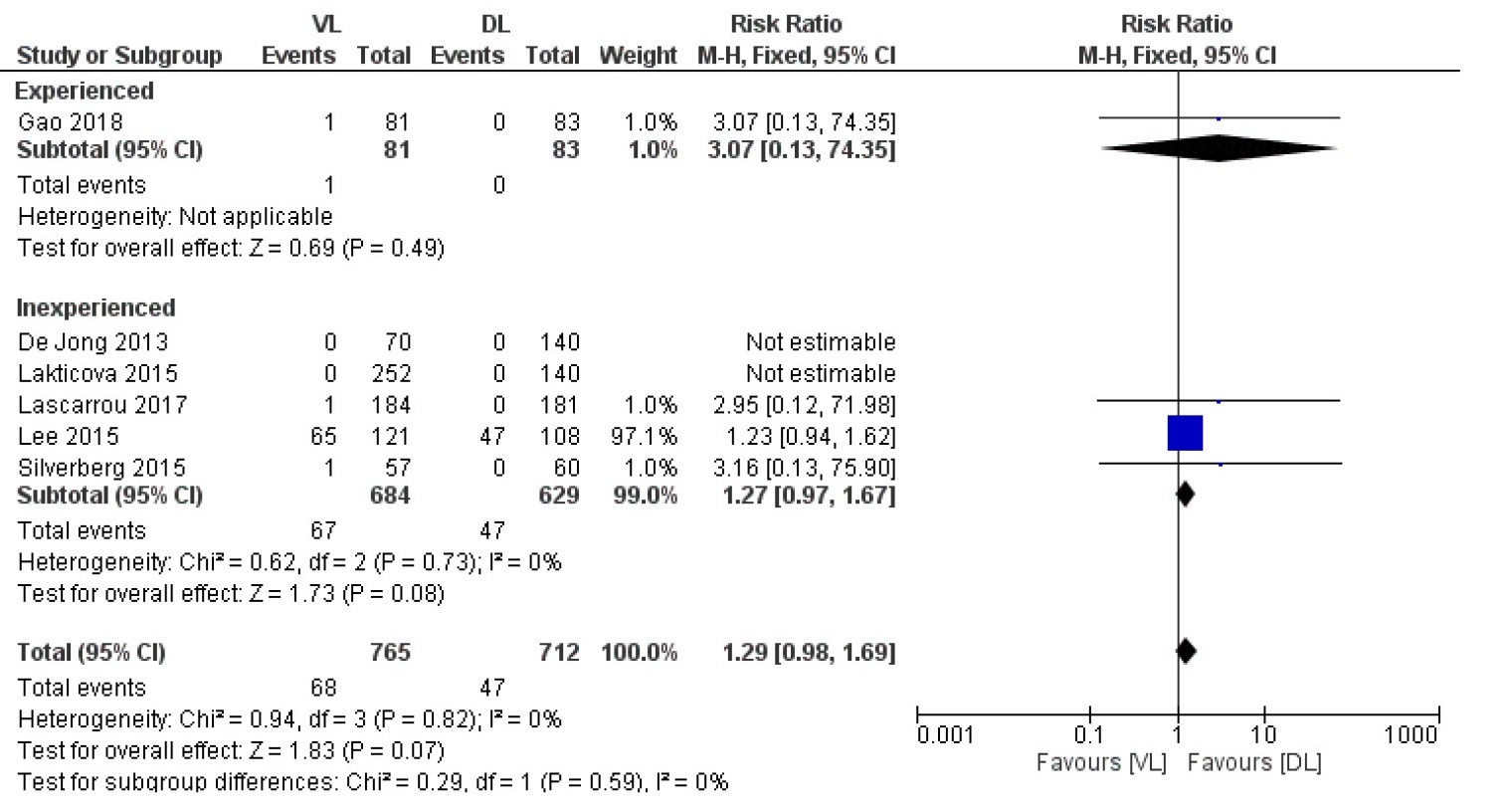


**Fig. S16** Forest plot for comparison of incidence of 24 h-mortality based on the experience of the operators between video laryngoscope (VL) and direct laryngoscope (DL). M-H, Mantel-Haenszel.


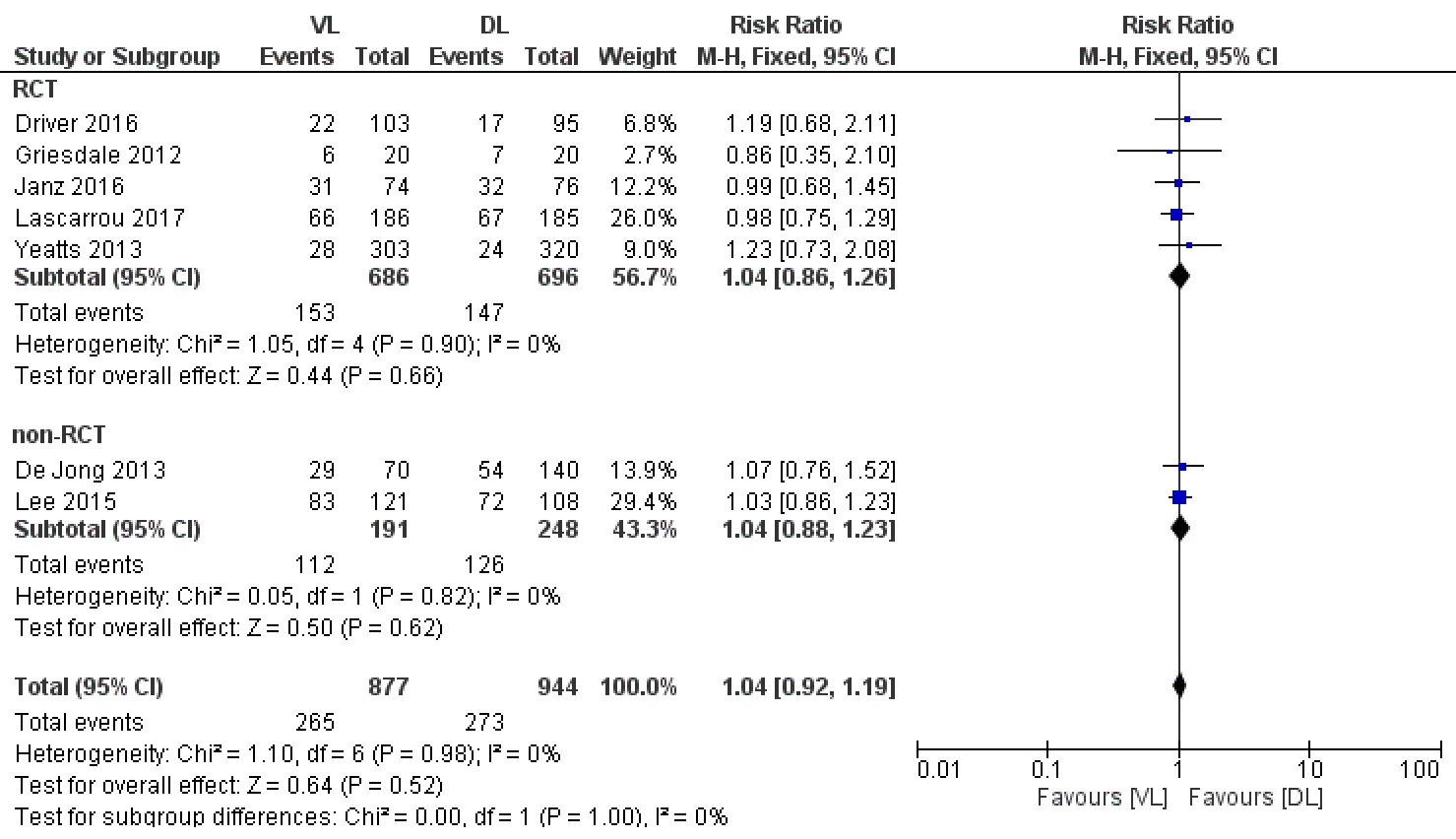


**Fig. S17** Forest plot for comparison of 28 d-mortality based on the type of studies between video laryngoscope (VL) and direct laryngoscope (DL). M-H, Mantel-Haenszel.


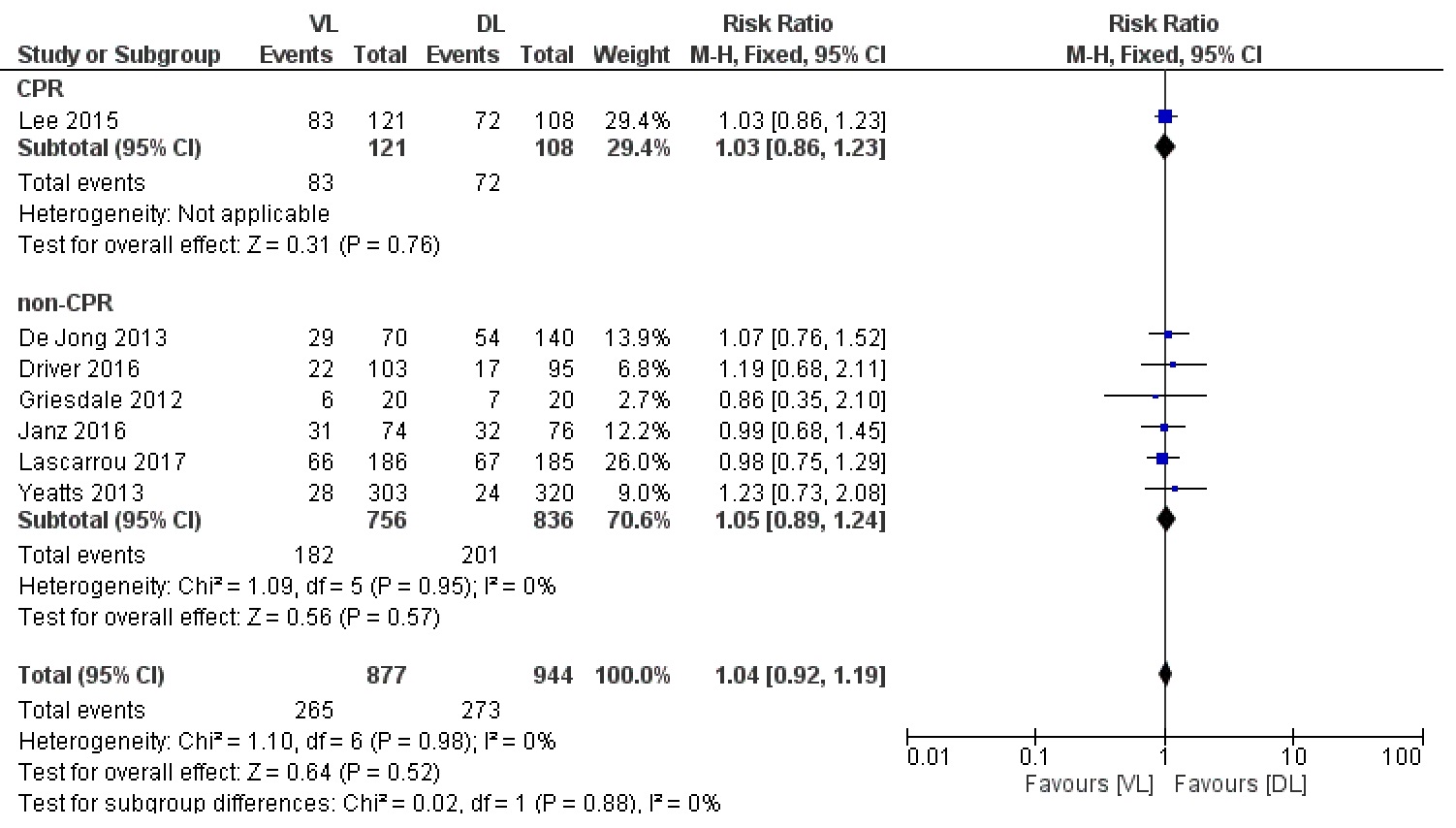


**Fig. S18** Forest plot for comparison of 28d-mortality based on whether a CPR study between video laryngoscope (VL) and direct laryngoscope (DL). M-H, Mantel-Haenszel.


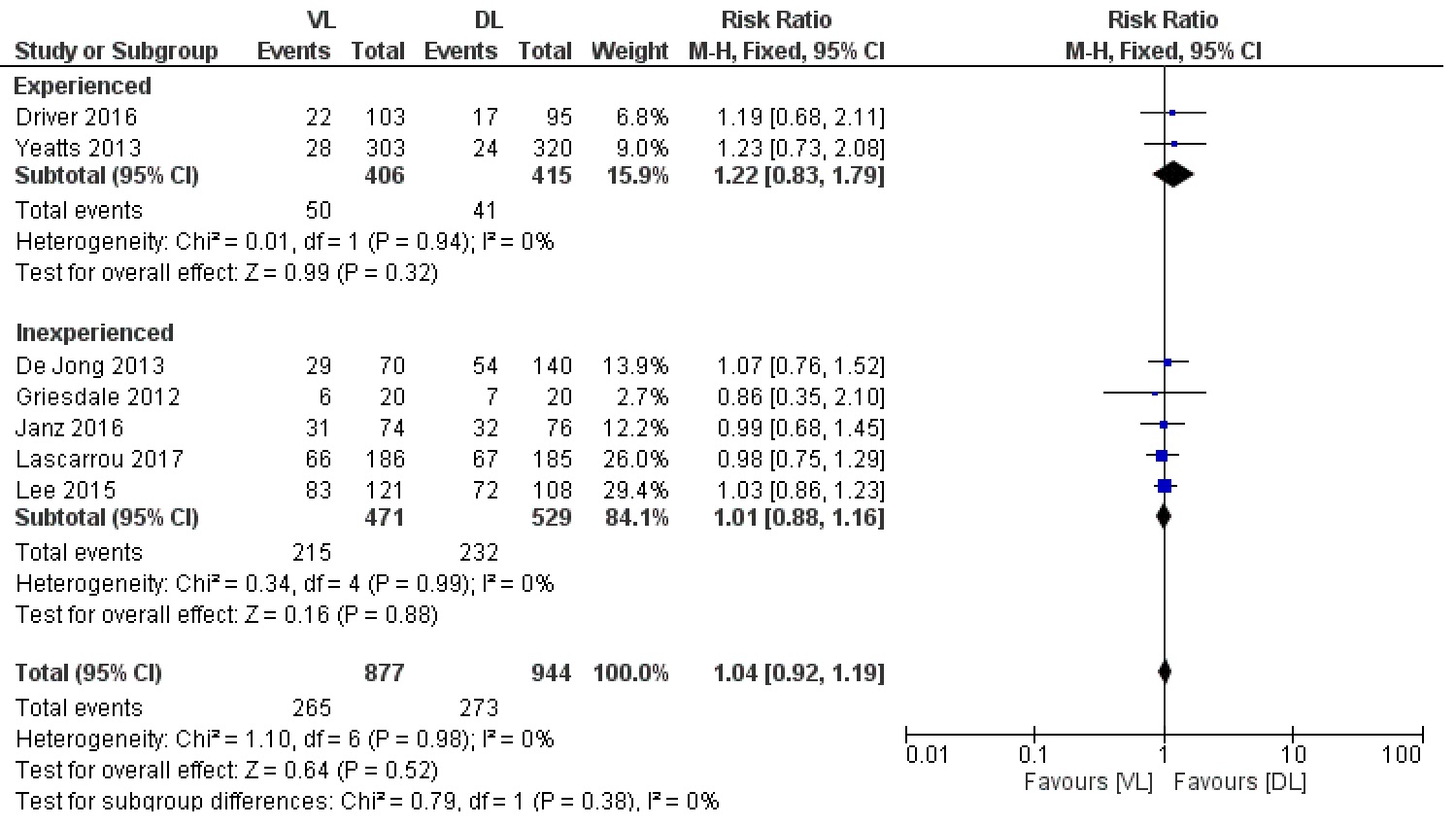


**Fig. S19** Forest plot for comparison of 28 d-mortality based on the experience of the operators between video laryngoscope (VL) and direct laryngoscope (DL). M-H, Mantel-Haenszel.


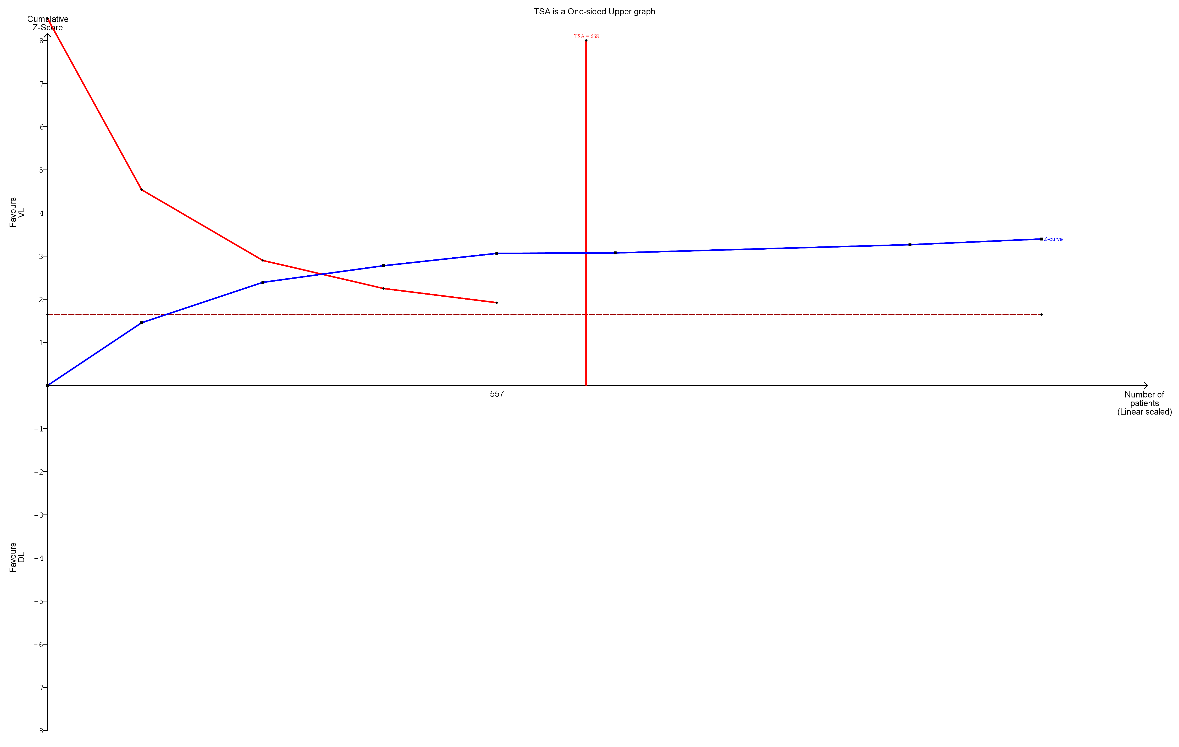


**Fig. S20 A**  TSA for rate of esophageal intubation based on 5% risk of type-1 error (one-sided upper), power 80%, low bias-based relative risk reduction of 69.1% and incidence in control arm of 4.9% with a model variance-based heterogeneity correction.


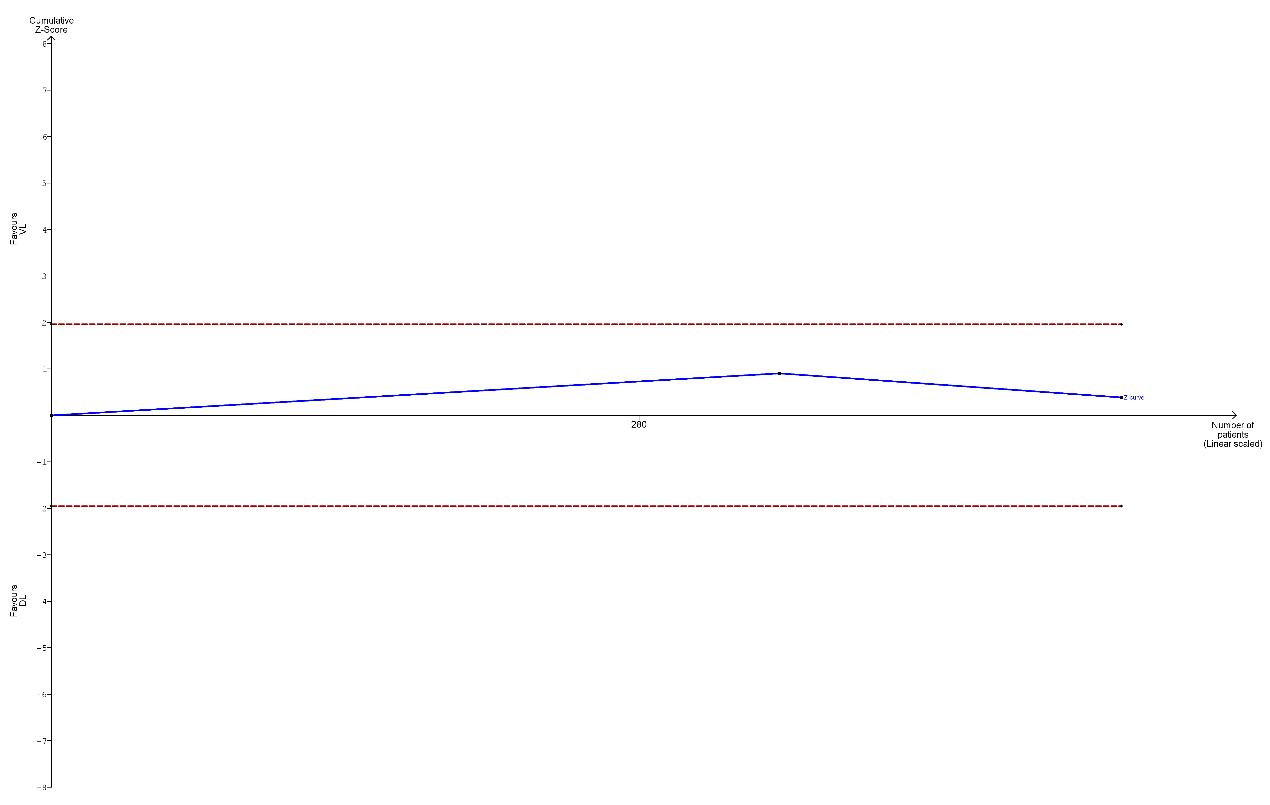


**Fig. S20B** TSA for incidence of hypoxemia based on 5% risk of type-1 error (two-sided), power 80%, low bias-based relative risk reduction of 8.89% and incidence in control arm of 12.1% with a model variance-based heterogeneity correction.


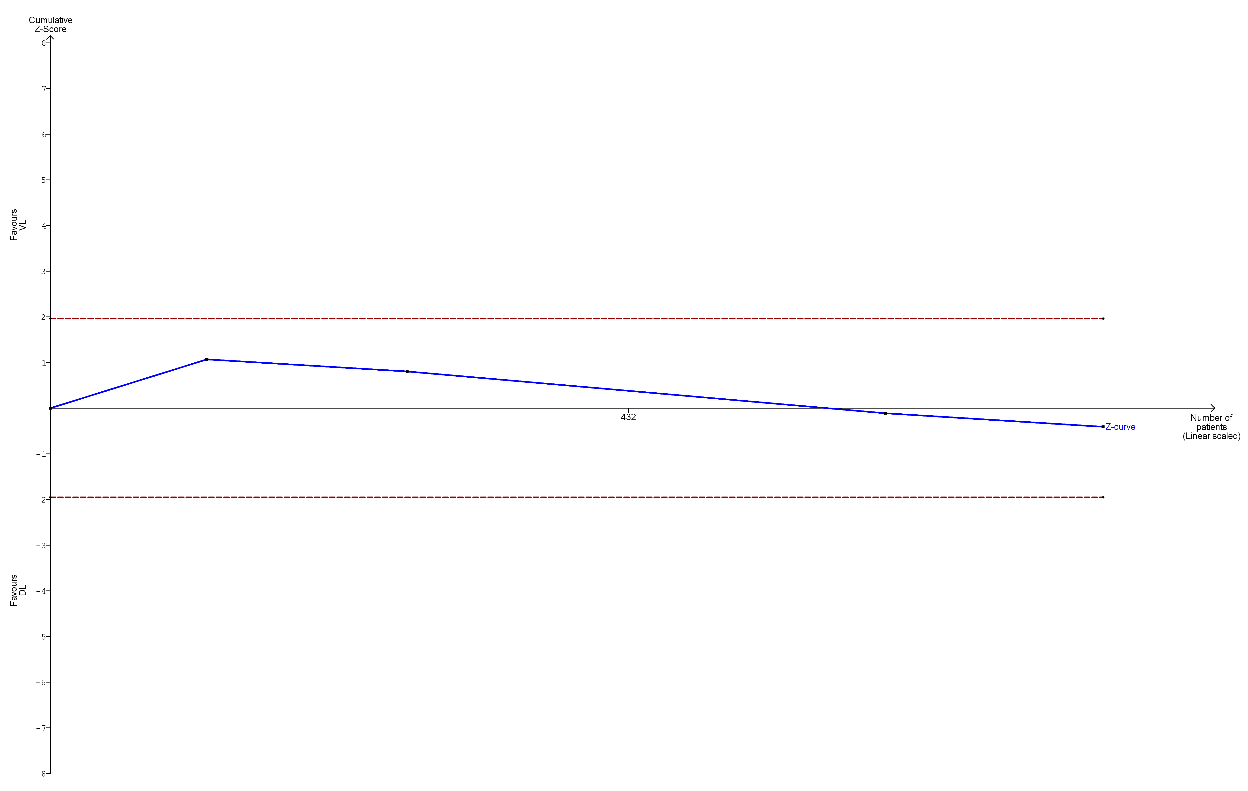


**Fig. S20C** TSA for incidence of severe hypoxemia based on 5% risk of type-1 error (two-sided), power 80%, low bias-based relative risk reduction of -29.5% and incidence in control arm of 6.0% with a model variance-based heterogeneity correction.

**
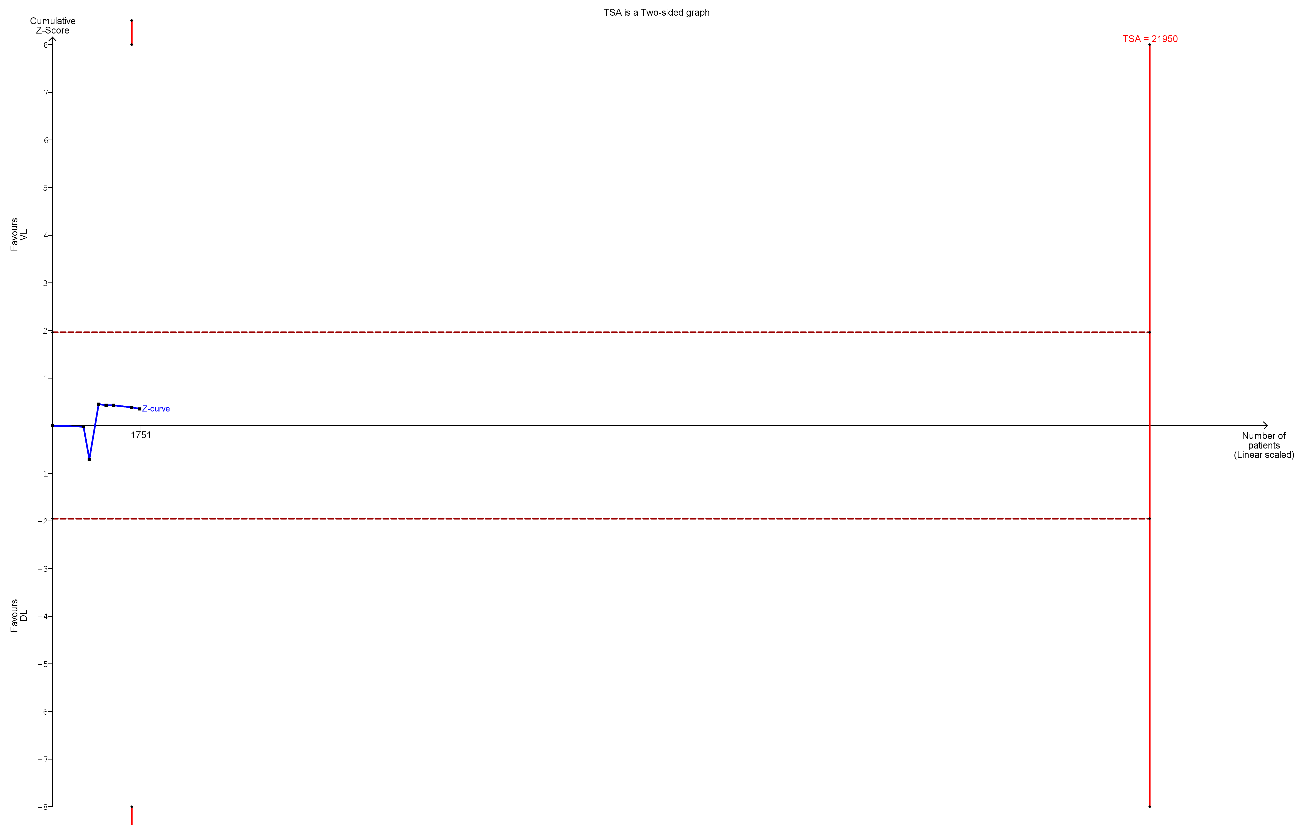
**

**Fig. S20D** TSA for the rate of aspiration based on 5% risk of type-1 error (two-sided), power 80%, low bias-based relative risk reduction of 21.32% and incidence in control arm of 2.7% with a model variance-based heterogeneity correction.


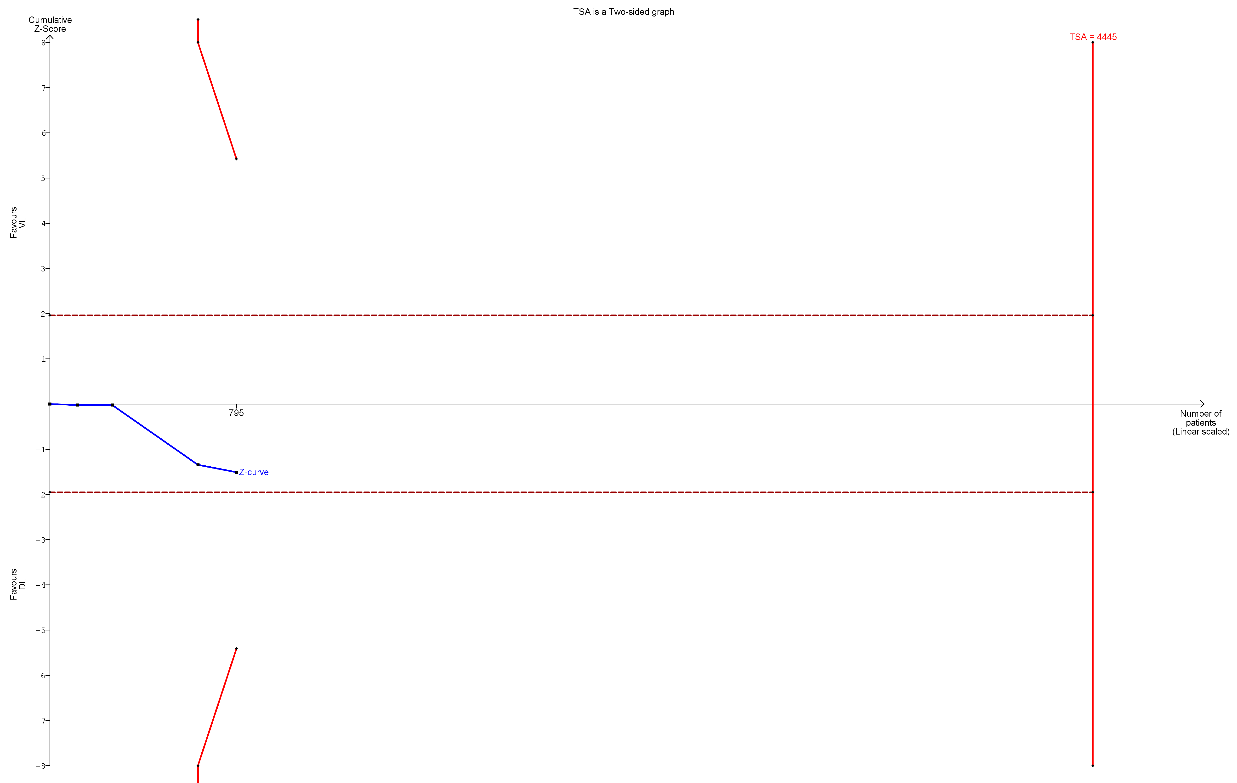


**Fig. S20E** TSA for incidence of new-onset cardiac arrest based on 5% risk of type-1 error (two-sided), power 80%, low bias-based relative risk reduction of -251.4% and incidence in control arm of 0.25% with a model variance‐based heterogeneity correction.


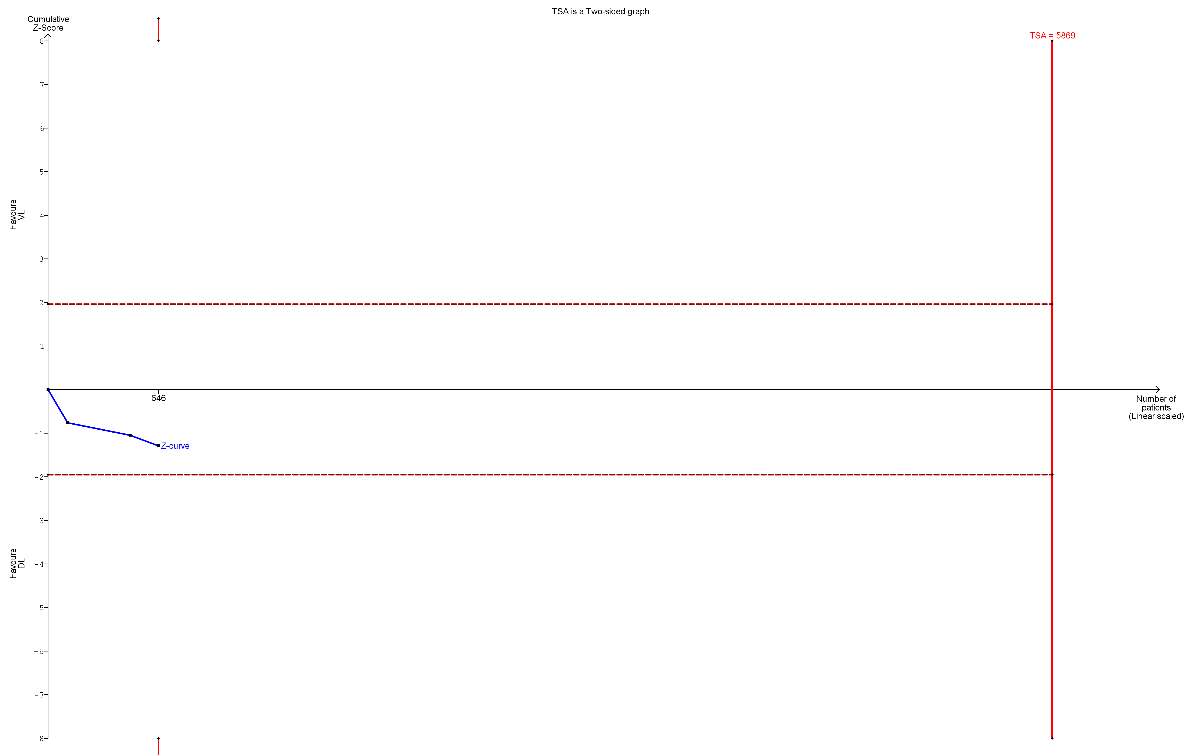


**Fig. S20F** TSA for 24 h-mortality based on 5% risk of type-1 error (two-sided), power 80%, low bias-based relative risk reduction of -402.0% and incidence in control arm of 0.1% (arbitrary set, 0 was not accepted) with a model variance-based heterogeneity correction.


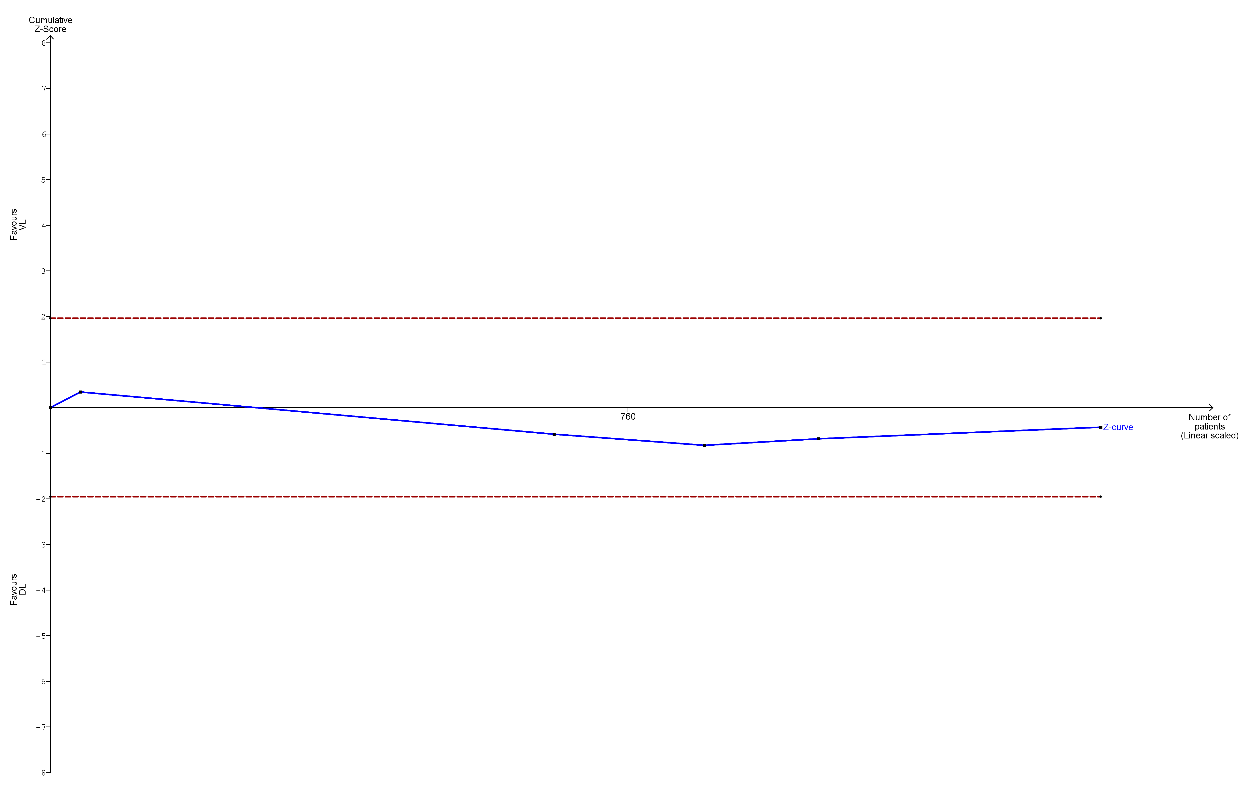


**Fig. S20G** TSA for 28 d-mortality based on 5% risk of type-1 error (two-sided), power 80%, low bias-based relative risk reduction of -4.31% and incidence in control arm of 21.1% with a model variance-based heterogeneity correction.
