# Supplementary material for: Comparison of adverse events between video and direct laryngoscopes for tracheal intubations in emergency department and ICU patients–a systematic review and meta-analysis
Source: Scand J Trauma Resusc Emerg Med. 2020 Feb 7;28:10. doi: 10.1186/s13049-020-0702-7 (PMC7006069; doi:10.1186/s13049-020-0702-7)
Supplement: Supplementary file 2 — Additional file 2: Tables S1-S2. The GRADE for all adverse events. The TSA for all adverse events from randomized controlled trials. [file 13049_2020_702_MOESM2_ESM.docx]

**Table S1 The quality of evidence for different adverse events**

| **Quality assessment** | | | | | | | **Number of patients** | | **Effect** | | **Quality** | **Importance** |
| --- | --- | --- | --- | --- | --- | --- | --- | --- | --- | --- | --- | --- |
|  |  |  |  |  |  |  |  |  |  |  |  |  |
| **No of studies** | **Design** | **Risk of bias** | **Inconsistency** | **Indirectness** | **Imprecision** | **Other considerations** | **VL** | **DL** | **Relative risk**  **(95% CI)** | **Absolute** |  |  |
| **Rate of esophageal intubation-RCTs** | | | | | | | | | | | | |
| 7 | RCTs | no serious | no serious | no serious | no serious | strong association^1^ | 7/616  (1.1%) | 30/616  (4.9%) | RR 0.27 (0.13 to 0.57) | 36 fewer per 1000 (from 21 fewer to 42 fewer) | ⊕⊕⊕⊕ High | Critical |
|  |  |  |  |  |  |  |  | 5.3% |  | 39 fewer per 1000 (from 23 fewer to 46 fewer) |  |  |
| **Rate of esophageal intubation - non-RCT** | | | | | | | | | | | | |
| 11 | observational studies | serious^2^ | serious^3^ | no serious | no serious | strong association^1^ | 56/4212  (1.3%) | 301/5743  (5.2%) | RR 0.24 (0.18 to 0.32) | 40 fewer per 1000 (from 36 fewer to 43 fewer) | ⊕OOO Very low | Critical |
|  |  |  |  |  |  |  |  | 5.2% |  | 40 fewer per 1000 (from 35 fewer to 43 fewer) |  |  |
| **Incidence of hypoxemia - RCTs** | | | | | | | | | | | | |
| 2 | RCTs | no serious | no serious | no serious | serious^4^ | none | 28/254  (11%) | 31/256  (12.1%) | RR 0.91 (0.56 to 1.47) | 11 fewer per 1000 (from 53 fewer to 57 more) | ⊕⊕⊕O Moderate | Important |
|  |  |  |  |  |  |  |  | 12.8% |  | 12 fewer per 1000 (from 56 fewer to 60 more) |  |  |
| **Incidence of hypoxemia - non-RCTs** | | | | | | | | | | | | |
| 2 | observational studies | serious^2^ | serious^3^ | no serious | serious^4^ | none | 166/790  (21%) | 76/248  (30.6%) | RR 0.83 (0.66 to 1.05) | 52 fewer per 1000 (from 104 fewer to 15 more) | ⊕OOO Very low | Important |
|  |  |  |  |  |  |  |  | 31.1% |  | 53 fewer per 1000 (from 106 fewer to 16 more) |  |  |
| **Incidence of Severe Hypoxemia - RCT** | | | | | | | | | | | | |
| 4 | RCTs | no serious | no serious | no serious | no serious | none | 26/388  (6.7%) | 24/399  (6%) | RR 1.11 (0.66 to 1.87) | 7 more per 1000 (from 20 fewer to 52 more) | ⊕⊕⊕⊕ High | Important |
|  |  |  |  |  |  |  |  | 5.4% |  | 6 more per 1000 (from 18 fewer to 47 more) |  |  |
| **Incidence of severe hypoxemia - non-RCT** | | | | | | | | | | | | |
| 4 | observational studies | serious^2^ | no serious | no serious | no serious | none | 62/509  (12.2%) | 46/443  (10.4%) | RR 1.13 (0.78 to 1.64) | 13 more per 1000 (from 23 fewer to 66 more) | ⊕OOO Very low | Important |
|  |  |  |  |  |  |  |  | 10.1% |  | 13 more per 1000 (from 22 fewer to 65 more) |  |  |
| **Incidence of aspiration - RCT** | | | | | | | | | | | | |
| 7 | RCTs | no serious | no serious | no serious | no serious | none | 22/869  (2.5%) | 24/882  (2.7%) | RR 0.9 (0.52 to 1.58) | 3 fewer per 1000 (from 13 fewer to 16 more) | ⊕⊕⊕⊕ High | Important |
|  |  |  |  |  |  |  |  | 2.2% |  | 2 fewer per 1000 (from 11 fewer to 13 more) |  |  |
| **Incidence of aspiration - non-RCT** | | | | | | | | | | | | |
| 6 | observational studies | serious^2^ | no serious | no serious | no serious | none | 50/1589  (3.1%) | 49/1294  (3.8%) | RR 0.8 (0.53 to 1.21) | 8 fewer per 1000 (from 18 fewer to 8 more) | ⊕OOO Very low | Important |
|  |  |  |  |  |  |  |  | 1.7% |  | 3 fewer per 1000 (from 8 fewer to 4 more) |  |  |
| **Incidence of new-onset cardiac arrest - RCT** | | | | | | | | | | | | |
| 4 | RCTs | no serious | no serious | no serious | very serious^5^ | none | 6/396  (1.5%) | 1/399  (0.3%) | RR 3.51 (0.73 to 16.92) | 6 more per 1000 (from 1 fewer to 40 more) | ⊕⊕OO Low | Important |
|  |  |  |  |  |  |  |  | 0% |  | - |  |  |
| **Incidence of new-onset cardiac arrest - non-RCT** | | | | | | | | | | | | |
| 3 | observational studies | serious^2^ | serious^3^ | no serious | very serious^5^ | none | 5/1096  (0.5%) | 5/542  (0.9%) | RR 0.86 (0.27 to 2.7) | 1 fewer per 1000 (from 7 fewer to 16 more) | ⊕OOO Very low | Important |
|  |  |  |  |  |  |  |  | 0.7% |  | 1 fewer per 1000 (from 5 fewer to 12 more) |  |  |
| **24 h-mortality – RCT** | | | | | | | | | | | | |
| 3 | RCTs | no serious | no serious | no serious | very serious^5^ | none | 3/322  (0.9%) | 0/324  (0%) | RR 3.06 (0.49 to 19.25) | - | ⊕⊕OO Low | Important |
|  |  |  |  |  |  |  |  | 0% |  | - |  |  |
| **24 h-mortality - non-RCT** | | | | | | | | | | | | |
| 3 | observational studies | serious^2^ | no serious | no serious | serious^4^ | none | 65/443  (14.7%) | 47/388  (12.1%) | RR 1.23 (0.94 to 1.62) | 28 more per 1000 (from 7 fewer to 75 more) | ⊕OOO Very low | Important |
|  |  |  |  |  |  |  |  | 0% |  | - |  |  |
| **28 d-mortality - RCT** | | | | | | | | | | | | |
| 5 | RCTs | no serious | no serious | no serious | no serious | none | 153/686  (22.3%) | 147/696  (21.1%) | RR 1.04 (0.86 to 1.26) | 8 more per 1000 (from 30 fewer to 55 more) | ⊕⊕⊕⊕ High | Important |
|  |  |  |  |  |  |  |  | 35% |  | 14 more per 1000 (from 49 fewer to 91 more) |  |  |
| **28 d-mortality - non-RCT** | | | | | | | | | | | | |
| 2 | observational studies | serious^2^ | no serious | no serious | serious^4^ | none | 112/191  (58.6%) | 126/248  (50.8%) | RR 1.04 (0.88 to 1.23) | 20 more per 1000 (from 61 fewer to 117 more) | ⊕OOO Very low | Important |
|  |  |  |  |  |  |  |  | 52.6% |  | 21 more per 1000 (from 63 fewer to 121 more) |  |  |

^1^ RR<0.5; ^2^ Observational studies; ^3^ Moderate heterogeneity (P < 0.1; I2 < 80%); ^4^ Few participants and few events; ^5^ Few participants and few events with wide confidence interval

**Table S2 Summary of TSA for all adverse events**

| **Outcomes** | **Boundary type** | **Type-1 error** | **Power** | **RRR^*^** | **Incidence in control arm** | **Heterogeneity correction** | **Information size** | **Conclusions** |
| --- | --- | --- | --- | --- | --- | --- | --- | --- |
| Rate of esophageal intubation | One-sided upper | 5% | 80% | 69.1% | 4.9% | model variance‐based | 668 | Conclusive: the cumulative z‐curve (blue line) crossed the boundary of required information size and trial sequential analysis monitoring boundary for favoring VL. |
| Incidence of hypoxemia | two-sided | 5% | 80% | 8.9% | 12.1% | model variance‐based | 27,519 | Inconclusive: boundary TSA is ignored due to too little information size. |
| Incidence of severe hypoxemia | two-sided | 5% | 80% | -29.5% | 6.0% | model variance‐based | 16,602 | Inconclusive: boundary TSA is ignored due to too little information |
| Incidence of aspiration | two-sided | 5% | 80% | 21.3% | 2.7% | model variance‐based | 21,950 | Inconclusive: the cumulative z‐curve (blue line) did not cross the boundary of required information size and trial sequential analysis monitoring boundary. |
| Incidence of new-onset cardiac arrest | two-sided | 5% | 80% | -251.4% | 0.25% | model variance‐based | 4,445 | Inconclusive: the cumulative z‐curve (blue line) did not cross the boundary of required information size and trial sequential analysis monitoring boundary. |
| Mortality-24 h | two-sided | 5% | 80% | -402.0% | 0.1%^**^ | model variance‐based | 5,869 | Inconclusive: the cumulative z‐curve (blue line) did not cross the boundary of required information size and trial sequential analysis monitoring boundary. |
| Mortality-28 d | two-sided | 5% | 80% | -4.3% | 21.1% | model variance‐based | 64,106 | Inconclusive: boundary TSA is ignored due to too little information |

^*^ **RRR:** relative risk reduction; ^**^This is arbitrary set. The actual data is 0, which is not accepted when calculating the information size needed.
